# The impact of intersectional racial and gender biases on minority female leadership over two centuries

**DOI:** 10.1038/s41598-023-50392-x

**Published:** 2024-01-02

**Authors:** Ganna Pogrebna, Spyros Angelopoulos, Immaculate Motsi-Omoijiade, Alexander Kharlamov, Nataliya Tkachenko

**Affiliations:** 1 Artificial Intelligence and Cyber Futures Institute, Charles Sturt University, Bathurst, NSW 2795 Australia; 2 Discipline of Business Analytics, The University of Sydney Business School, University of Sydney, Abercrombie Building H70, Corner Abercrombie Street and Codrington Street, Darlington, NSW 2006 Australia; 3 The Alan Turing Institute, 96 Euston Rd, Kings Cross, London, NW1 2DB UK; 4 Durham University Business School, Mill Hill Lane, Durham, DH1 3LB UK; 5 RAND Corporation, Westbrook Centre, Milton Rd, Cambridge, CB4 1YG UK; 6 Cranfield School of Management, Cranfield University, College Rd, Cranfield, Wharley End, Bedford, MK43 0AL UK; 7 Cambridge Judge Business School, University of Cambridge, Trumpington St, Cambridge, CB2 1AG UK

**Keywords:** Human behaviour, Social evolution

## Abstract

This study scrutinizes the enduring effects of racial and gender biases that contribute to the consistent underrepresentation of minority women in leadership roles within American private, public, and third sector organizations. We adopt a behavioural data science approach, merging psychological schema theory with sociological intersectionality theory, to evaluate the enduring implications of these biases on female leadership development using mixed methods including machine learning and econometric analysis. Our examination is concentrated on Black female leaders, employing an extensive analysis of leadership rhetoric data spanning 200 years across the aforementioned sectors. We shed light on the continued scarcity of minority female representation in leadership roles, highlighting the role of intersectionality dynamics. Despite Black female leaders frequently embracing higher risks to counter intersectional invisibility compared to their White counterparts, their aspirations are not realized and problems not solved generation after generation, forcing Black female leaders to concentrate on the same issues for dozens and, sometimes, hundreds of years. Our findings suggest that the compound influence of racial and gender biases hinders the advancement of minority female leadership by perpetuating stereotypical behavioral schemas, leading to persistent discriminatory outcomes. We argue for the necessity of organizations to initiate a cultural transformation that fosters positive experiences for future generations of female leaders, recommending a shift in focus from improving outcomes for specific groups to creating an inclusive leadership culture.

## Introduction

Despite rising awareness of female minority leadership due to notable diversity gaps^1^ and its pivotal role in strategic management^2,3^, practical advancements lag behind theoretical progress^4,5^, sustaining the underrepresentation of Black female leaders as a vital concern^6,7^. The statistics reflect this disparity: in 2021, White women held 32.6% of managerial positions in the US, while Black women occupied only 4.3% of such positions^8^. Additionally, there is a lack of Black women CEOs in Fortune 500 companies. In 2023, women led 10.4% of the Fortune 500 companies. Yet, only two of them had Black (more specifically, African American) heritage^9^. Despite modest progress in the representation of women in senior leadership positions, Black women continue to face unique challenges, being promoted at a slower pace and significantly underrepresented in top leadership roles^10^. The underrepresentation of minority women in leadership positions has been shown to hinder organizational success, and minority individuals who have had leadership opportunities were found to be more productive and efficient in their roles than their majority counterparts^11,12^.

The exploration of leadership outcomes for racial majorities versus minorities in underrepresented groups has predominantly been approached through the lens of intersectionality theory^13,14^. This theory examines the combined effects of “racist and gendered stereotypes” on female minority leaders who face compounded discrimination due to their association with both an underrepresented gender group and a specific ethnic minority^15^. However, the lack of progress in female minority leadership can be partly attributed to the limitations of the intersectionality approach and its policy recommendations. One of these limitations is the difficulty in measuring and defining intersectional effects, as the narrative-based nature of intersectionality theory hinders consensus on standardized measurement approaches^13^. Additionally, the static nature of intersectional effects overlooks the dynamic and complex historical development of multifaceted identities, often treating race and gender as fixed constructs^16^. Consequently, the literature has primarily focused on the bicultural competencies or leadership styles of ethnic minority females, with empirical studies relying predominantly on qualitative data due to the challenge of quantifying intersectionality^14,17,18^. As a result, intersectional effects are often derived from relatively small data samples, limiting generalizability.

To address these gaps, there is a need for a quantitatively testable theory that captures the dynamic intersectional effects and their influence on the development of female minority leaders. In this study, we propose a new theoretical approach, grounded in the interdisciplinary field of behavioural data science, which considers the intersectional impact of racial and gender stereotyping over time. Our approach combines hypothesis-driven views from intersectionality theory and schema theory, along with a data-driven approach that allows for quantitative testing using machine learning, statistical, and econometric techniques. Building on past minority leadership frameworks^19–21^, our approach diverges from previous research. While earlier models viewed gender and race as static factors in shaping female minority leadership, we see them as dynamic elements, continuously influencing leaders’ experiences and actions over time.

Historically, in the annals of American history, Black women have faced multi-dimensional challenges due to the intersections of their race and gender. The concept of intersectionality^22^ offers an analytical lens to understand the unique experiences Black women undergo, especially when they hold authoritative roles in organizations dominated by White individuals. These challenges are not merely the sum of racial and gender-based prejudices; they intertwine in a manner that shapes the distinct social realities of these women. The experiences of Black women cannot be neatly compartmentalized into separate spheres of race, gender, and social class^23^. Instead, these spheres overlap, painting a picture that traditional feminist discourse often overlooks^12^. This intersectional approach is vital, given the long history of Black women being positioned as outsiders within dominant organizational cultures, both in academia and business. As they climb the leadership ladder, they grapple with unique obstacles that stem from historical stereotypes and prejudices. The dual challenges of racism and sexism mean that their leadership journey often involves navigating a “double jeopardy”. This double bind of facing discrimination due to their racial identity and their gender identity underscores the resilience and determination of Black women leaders.

Women collectively face challenges like sexism and discrimination^24^. Yet, Black women also grapple with racism’s added burden. Their dual challenges, rooted in gender and race, amplify the hurdles they confront in leadership roles. Using an example of African-American ethnic minority, previous literature demonstrated that Black women’s unique disadvantage arises from the intersectionality of race and gender, deeply tied to historical events such as slavery^25^. They face persistent stereotypes due to a legacy of slavery and systemic biases^26^. The combined racial and gender bias often leads to their isolation in workplaces^27^.They encounter heightened expectations, compelling them to consistently excel in their fields in pursuit of acknowledgment^24^. Their journey in leadership underscores their tenacity and drive, as they uniquely overcome numerous challenges distinct from those, encountered by their counterparts^12^.

Our proposed approach focuses on the three stages of female minority leadership:

1. *Identification Stage* leaders possess certain intersectional priors, such as being female and belonging to an ethnic minority group. Based on these priors, family development context, individual characteristics, and knowledge of previous leaders with similar priors, each leader determines their risk tolerance and self-selects into a leadership role within specific fields.
2. *Progression Stage* leaders interact with the world, gain experiences, and update their behavioral schemas. Behavioral schemas help individuals process information from new experiences and adapt to change. Goals are set, and learning occurs through successes and failures. Rigidity in behavioral schemas persists when goals and priorities are not achieved, whereas schema updating occurs when goals are attained. During this stage, leaders gain recognition from followers within their respective fields.
3. *Achievement Stage* leaders gain societal recognition as prominent figures in leadership. Achievement is viewed as a direct outcome of the previous two stages.

By considering the intersectional effects of gender and race and understanding how they influence risk attitudes in the identification stage and modify behavioral schemas in the progression stage, we gain a dynamic perspective on the determinants of diversity gaps in female leadership. Although our theory can be applied to individual leaders and groups of leaders, our empirical focus is on groups of female leaders with different intersectional priors, specifically comparing Black female leaders with those from the White majority group. We collect demographic, background, and speech data for 757 female leaders spanning a time period from the 19th century to the end of 2019, of which 608 were American. The end of 2019 is chosen as a cut-off date to avoid potential COVID-19 bias in leadership speeches from 2020 onwards. All women in our sample are recognized by both the college of experts and by the general public as influential leaders in one or more fields, ensuring a similar level of high leadership achievement.

The remainder of this paper is structured as follows. In section "Methods" describes methods used to conduct this research, justifies our theoretical assumptions, formulates hypotheses, and highlights contributions of our research. In section "Justification of assumptions" presents results of the empirical test of our model. Finally, we conclude by discussing the general implications of our study.

## Methods

**Figure 1:**
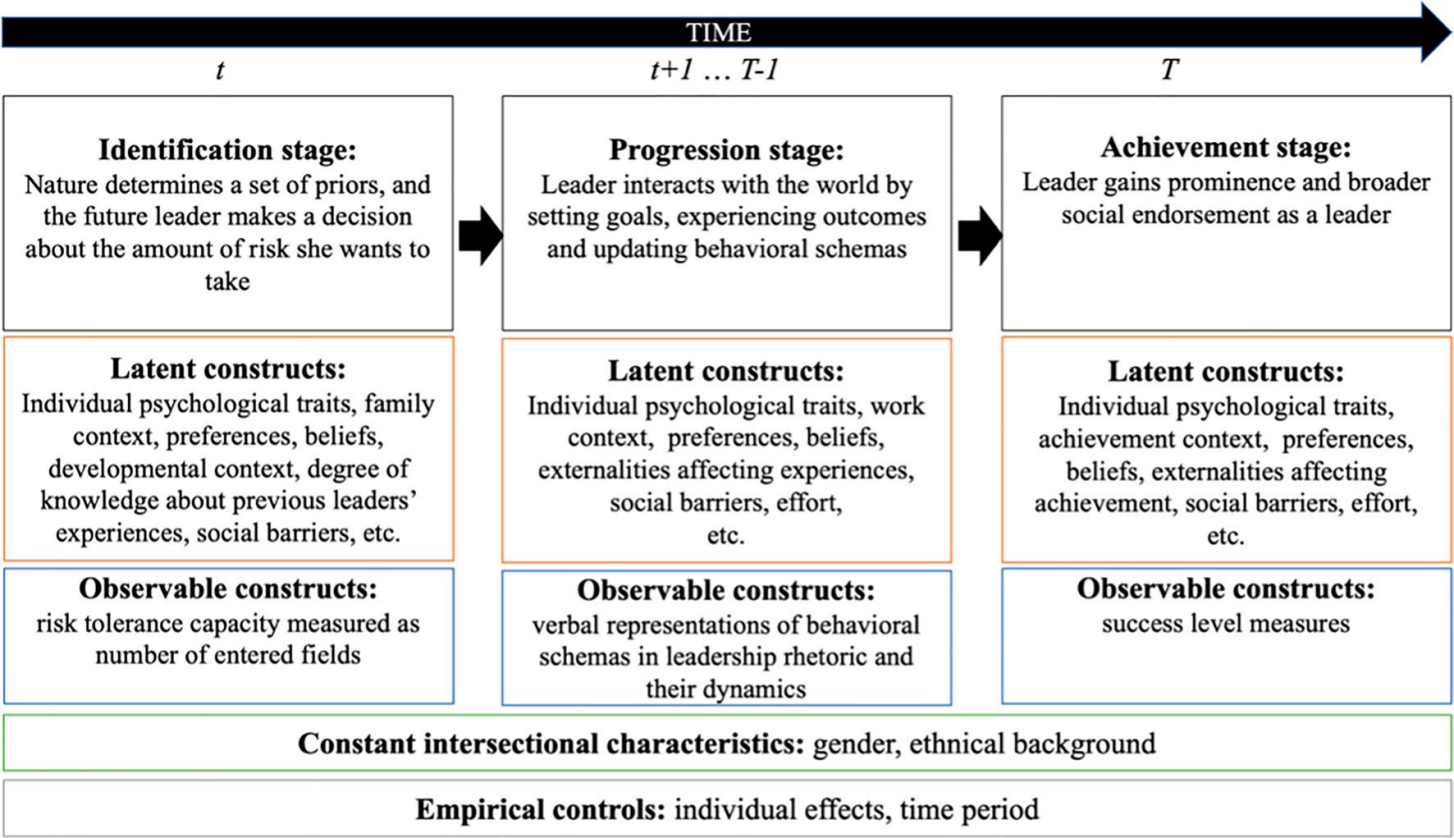
The Structure of Conceptual Behavioural Data Science Model and Empirical Strategy. *Note* The figure demonstrate a 3-stage leadership model describing Identification, Progression, and Achievement stages of leader’s development. It also explains observable and latent constructs associated with each stage.

Our basic conceptual model is based on the cross-disciplinary behavioural data science approach. Consider a 3-stage dynamic problem with the timeline depicted on Fig. 1. The leader goes through 3 stages in her development: Identification, Progression, and Achievement.

During the Identification stage, the “Nature” determines a set of priors, which it assigns to the future leader. The future leader considers the amount of risk she wants to take in her leadership career by deciding on the number of areas or fields she wants to enter. Since each entry is inherently costly, requiring the leader to take on multiple visible responsibilities, and, therefore, it is also risky: the higher is the number of entered fields, the higher is the risk tolerance capacity of the future leader. The number of entered fields is observable (e.g., it is easy to collect information on whether a particular leader decided to concentrate on one particular field such as law or decided to diversify into several fields by entering the field of law and, in parallel, engaging into activism and then politics). Yet, the decision to enter multiple fields may result from multiple factors such as suffering from the intersectional invisibility (i.e., being overlooked as a non-prototypical representative of a cultural group) due to gender and racial stereotyping^14^, as well as many latent factors such as individual psychological characteristics, family context, preferences, beliefs, developmental opportunities, etc.^20,21^. It also may be the case that the future leaders consider the experience of previous leaders with similar cultural characteristics (i.e., they may have role models from their cultural group), yet, the degree of knowledge or awareness about these experiences as well as the way in which future leaders perceive these experiences are unobservable. Nevertheless, all these constructs may impact a leader’s decision-making process, which is why it is important to account for these possible effects in our empirical analysis.

In the Progression stage, the leader engages into leadership activity in organizational context through translating her set of leadership priorities by verbalizing her behavioral schemas. These schemas help the leader to set goals for herself as well as her followers and update the schemas once the goals are achieved. The verbalized schemas are measurable by considering the topics, on which leaders concentrate in their rhetoric as well as the speed with which these topics change over time. Apart from the gender and racial stereotyping, the leaders may be affected by a number of individual and contextual observable as well unobservable factors, which we are accounting for in our empirical analysis by controlling for individual effects as well as time effects.

Finally, in the Achievement stage, the leader gains prominence and is accepted by the broader society (not just by her immediate following within her organization) as a leader. At this stage, the observable construct is usually success level, which may have different measures from specific variables (such as the level of corporate profit, shareholder value, return on investment, etc.). It may also be as simple as a binary variable (where success is assumed to be 1 and failure is depicted as 0) or continuous (where success is distributed between 0 and 1). Similarly to previous stages, a number of important latent effects need to be taken into account in the empirical analysis. In our empirical strategy (described below) we assume that all leaders in our dataset are equally successful (in other words, we keep the leadership success level constant and assume it to be equal to 1 as this assumption most appropriately reflects our dataset).

To give a specific example of Black female leader, Fannie Lou Hamer’s trajectory from a Mississippi sharecropper to a nationally recognized leader epitomizes the three-stage leadership model^28^. Initially, during the Identification stage, Hamer confronted racial injustices and risked her safety to register to vote, later diversifying into civil rights activism with the SNCC and CORE. In the Progression stage, her grassroots leadership style and impactful speeches, especially during the 1964 Democratic National Convention, illuminated the barriers faced by Black voters. Transitioning to the Achievement stage, Hamer shifted focus to economic disparities, co-founding the Freedom Farm Cooperative and advocating policies promoting Black economic upliftment, education, and health, solidifying her legacy in American civil rights history.

Considering the three stages described above, our basic behavioural data science model can be specified as follows. Consider an individual leader *k*, characterized by a set of cultural and socio-demographic priors. We concentrate on female leaders with different racial and ethnic backgrounds, hence, we assume that $k\in \{i,j\}$, i.e., $k=i$ if the female leader identifies herself with an ethnic minority and $k=j$ if she identifies herself with the ethnic majority. The leader’s success (achievement) is depicted by $s_k^T$, which depends on the leader’s risk tolerance capacity $r_k^t$ as well as her behavioral schema updating measure $\sigma _k$, capturing the relative importance of a particular topic of focus for the leader over time. This measure can be defined in many ways, but we use the average standard deviation of probabilistic measure of importance of all topics in leadership rhetoric over $N$ number of time periods (years) as specified in subsequent sections. In other words, $\sigma _k = \frac{1}{\eta } \sqrt{\left( \sum _{\tau =t+1}^{T-1} \left( e_k^{\tau }-\mu \right) ^2 \right) /N}$, where $e_k^\tau$ is each probabilistic value of topic importance from the pool of important topics considered in leadership rhetoric, $\mu$ is the pool mean, *N* is a number of time periods and $\eta$ is the number of important topics, which represent verbalized behavioral schemas of the leaders. Therefore, the leadership success (achievement) is determined by a combination of a leader’s ability to take risk (i.e., given by her risk tolerance capacity) combined with her experiences (i.e., experientially informed and constantly updating behavioral schemas) and is given by: $s_k^T=r_k^t+\sigma _k+\varepsilon$ where $\varepsilon$ is a normally distributed noise parameter, which captures any possible stochastic shocks to the leadership trajectory.

In understanding the leadership journey of Black women, it is imperative to consider the intersectionality of race and gender. Black women often face a unique set of challenges and experiences as they navigate leadership roles. This intersectionality does not simply mean they experience the additive challenges of being Black and being a woman, but rather, they face specific issues arising from the combination of these identities. For example, they might encounter racialized sexism or gendered racism in their roles. In our study, we specifically delved into these unique challenges and how they shape the leadership styles, strategies, and decisions of Black women.

We recognize that there is a significant difference between being a Black woman leader and being a Black woman leader who actively advocates for Black issues. While all Black women leaders bring their lived experiences into their leadership, not all choose or have the opportunity to center their leadership around advocacy for Black issues. Our research methodology differentiates between these two categories, ensuring we capture a holistic understanding of Black women’s leadership. We have ensured that our study both recognizes the diversity within Black women leaders and understands the specific nuances of those who are advocates for Black issues.

### Justification of assumptions

#### Concentration on gender and ethnicity

Even though intersectional stereotyping in organizations may arise from multiple intersectional systems in the society (race, gender, socio-economic class, ability, age), we concentrate on the ethnic background and gender for several reasons. First, the impacts of gender and ethnic background are often overlooked compared to other combinations such as, e.g., gender and age, etc. As Crenshaw puts it using an example of Black female leaders, “Black women’s intersectional experiences of racism and sexism have been a central but forgotten dynamic in the unfolding of feminist and antiracist agendas...”^29^. Second, the effects of gender and race are difficult to measure due to the fact that, unlike many other factors, both gender and race involve self-attribution. Unlike other characteristics, that are easy to measure objectively (e.g., age), an individual needs to associate herself with women and with a particular minority group in order for intersectionality analysis to be valid. Yet, our unique dataset allows us to focus on leaders, who identify themselves as females and as representatives of a particular racial and ethnical background.

#### Stability of gender and ethnical background identification

Our model assumes that gender as well as racial and ethnical identification remain constant throughout all three stages of the leader’s development. In practice, this may not always be the case as gender, racial, and ethnical identification may change over time. For example, a particular leader with a mixed ethnical background, e.g., Austrian and Japanese, may first identify as an Austrian because she was raised by her family as an Austrian. Yet, later is life she may decide to identify herself as Japanese. The leader may also undergo a trans gender transition through her life, e.g., first being known as a man and later as a woman. Consider, for example, the Wachowskis—leaders in the film making and authors of the Matrix movies—who were first known as men and are currently known as trans women. These processes are rare and complex and, most importantly, their intersectional effects are not very well understood in the literature, which is why we concentrate on women leaders with stable gender, race, and ethnicity identification over time.

#### Separability of risk tolerance determination and experiences

Our model implies that the leader’s risk tolerance capacity determination and behavioral schemas updating occur in different stages of a leader’s development (i.e., are time-separable). Specifically, the leader first makes a decision about how many fields she wants to enter (determines her risk tolerance capacity) and then proceeds to having experiences in these fields, updating her view of the word (behavioral schemas) and verbalizing these schemas as they emerge and update. This, however, may not always be the case. For example, the leader may initially decide to enter only one field (e.g., law) and through her experiences then decide to later enter another field (e.g., politics). The time separability is not an important assumption for our model: in principle, the Identification and Progression stages can be happening at the same time as long as we acknowledge that leaders’ risk tolerance capacity determination and behavioral schemas as products of experiences are distinct components of the leader’s development. Considering risk tolerance updating as a part of behavioral schemas updating would be an interesting extension of our model, which does not consider how the updating is happening (i.e., in order to incorporate risk tolerance updating into a model one would first need to explain the exact mechanism of behavioral schemas updating, which is outside the scope of this paper). We discuss this as a limitation of our model in the concluding section of this paper and provide several suggestions about how the updating process could be modeled in the future studies. Nevertheless, recent evidence from studies on female leadership and female ethnical minority leadership supports the validity of the separability assumption. Using qualitative interviews, these studies find that women leaders often make decisions to have multiple roles, take on many responsibilities, and diversify their efforts across several fields prior to engaging in leadership experiences in order to tackle gender and racial discrimination or to become more visible^13,14,30,31^.

### Empirical methodology and testable hypotheses

Even though our model could be applied to the individual leaders, we are particularly interested in racial and ethnic minority female leaders as a group. Instead of looking at the difference in leadership success (achievement) given leader’s characteristics, our empirical strategy is to hold the level of success constant and explore the differences between risk tolerance capacity and experiential behavioral schemas of majority and minority female leaders. We focus on women who all achieved societal endorsement in the sense that they became widely known and accepted by people within and outside their organizations and fields as leaders. For simplicity, if 0 would constitute the failure to achieve societal acceptance as a leader and 1 would constitute success, we assume that in our sample all female leaders achieved $s_k^T = 1$ irrespective of their background. This means, that the success level of minority and majority leaders is the same, i.e., $s_i^T=s_j^T$. This implies that $r_i^t + \sigma _i + \varepsilon = r_j^t + \sigma _j + \varepsilon$ or, considering that $\varepsilon$ is normally distributed and does not depend on whether the leader is or is not a representative of the racial or ethnic minority: $r_i^t+\sigma _i=r_j^t+\sigma _j$. This allows us to formulate a number of testable hypotheses, as if $\sigma _i<\sigma _j$, $r_i^t$ should be greater than $r_j^t$. Alternatively, if $\sigma _i>\sigma _j$, $r_i^t$ should be lower than $r_j^t$. There is also a theoretical possibility that both risk tolerance capacity and experiences are the same for both majority and minority leaders, i.e. if $\sigma _i=\sigma _j$, then $r_i=r_j$. Since we observe that female minority leaders are disadvantaged compared to the female majority leaders^13^, we expect their leadership experiences to be a lot less positive than those of the female majority leaders, meaning that minority female leaders should update their behavioral schemas less often than majority female leaders. This implies that minority female leaders are forced to concentrate on the same priorities and verbalize the same behavioral schemas over multiple time periods, whereas majority female leaders are able to change their priorities often. Therefore, the standard deviation of the probabilistic measure of topic importance should be lower for the minority leaders than for the majority leaders. Hence, our first hypothesis could be formulated as follows.

#### Hypothesis 1

 Verbalized behavioral schemas of minority female leaders should be less time-variant and less dispersed than those of the majority female leaders.

This means that we should observe that $\sigma _i<\sigma _j$, i.e., the mean standard deviation of the topic probabilities (measured by the topic modelling in the leadership rhetoric) should be lower for the minority compared with the majority group. If minority leaders generally have less positive leadership experiences, this means that they should be compensating for the lack of positive progress in achieving their goals by taking more risk and concentrating on more fields than their majority counterparts. A recent longitudinal study approached 59 Black females twice over the course of 7 years and found through a series of qualitative interviews that these women tackled career challenges associated with intersectional invisibility by what we describe as a more risk-taking behavior^14^. Specifically, these women took on more responsibilities and engaged in larger number of visible leadership roles to progress in their careers. Importantly, the interview evidence suggests that these women first made the decisions about the number of roles and responsibilities and then proceeded to their experiences. Another study focused on minority female leaders in Pakistan, the United Kingdom as well as Brazil, who revealed through a set of qualitative interviews that they needed to deliberately plan to take more responsibility and engage in more roles before they could proceed to having leadership experiences^32^. Further studies have confirmed that the necessity for higher risk taking becomes apparent from an early age as future minority leaders are encouraged to respond to more opportunities by their families^30^ as well as opt for more visible and diverse set of responsibilities throughout the school year^31^. Therefore, our second hypothesis can be formulated as follows.

#### Hypothesis 2

 Minority female leaders tend to take more risk with their leadership careers than their majority counterparts.

Considering the lack of improvement in female minority leadership over time, our model also allows us to formulate the third hypothesis:

#### Hypothesis 3

Since experiences improve for majority, but not for minority female leaders over time, minority leaders (as a group) should take progressively more risk than majority leaders.

### Data mining and analysis

One of the main constructs in our behavioural data science model is the construct of a behavioral schema, which allows the leaders to comprehend, internalize, and understand their goals and priorities. These priorities need to be communicated to others and if they are achieved, the leader develops a new set of behavioral schemas, which, in turn, need to be communicated again. The extant psychological and leadership literature stresses the importance of effectively expressing and communicating behavioral schemas to others: leaders often verbalize schemas as strategic directions, values, and vision, to achieve development of organizations, and empowerment of followers^33–35^. Hence, leadership rhetoric is suggested as a good proxy to measure leaders’ dynamic behavioral schemas^36–39^. We concentrate on female leaders’ rhetoric and communication.

Our strategy is to concentrate on successful female leaders, who not only self-select into the leadership roles, but also achieve societal recognition as leaders. We use an inclusion into a leadership repository of high achievers as a proxy of success, which implies that women represented in these repositories are endorsed not only by their direct followers in organizations, but also by the broader society as being “worthy” of being included in the repository. We obtained data from two repositories: the Iowa State University Archives of Women’s Political Speech and the Gifts of Speech. Both repositories are hosted by universities and inclusion into the repositories is subject to expert review. The Iowa State University Archives of Women’s Political Speech is hosted by the Iowa State University, a public university in Ames, Iowa, and collects specimen of speech in English from prominent women who through their whole careers or at some point in their careers were leaders in activism, politics, civil service, public life, or assumed other leadership roles of power and public importance. The Gifts of Speech is hosted by Sweet Briar College, a private women’s college in Sweet Briar, Virgnia, and collects speech specimen in English from prominent female leaders representing a wide variety of fields. Though both repositories aim to suggest role models to the future generation of (female) leaders and a college of experts is consulted before including a particular individual into each of the repositories, the Iowa State repository includes women who are widely accepted as leaders by the American public (for example, prominent female American business women, lawyers, politicians, activists, journalists, etc., are included in the repository), the Gifts of Speech repository appears to apply additional success criteria such as winning an important global award such as the Nobel Prize or an well-known specialized prize such as Fields Medal, etc.

Our focus on Black women leaders who are widely recognized by the general public was chosen for its accessibility and relatability. However, we acknowledge the potential limitations inherent in focusing solely on these leaders. Historical and contemporary structures may determine which Black women are allowed or chosen to be in the limelight. For instance, certain personality traits, leadership styles, or even appearances might be deemed more “palatable” or “acceptable” by mainstream society, thereby allowing some Black women to rise to prominence over others. In our analysis, we aim to understand the systemic, societal, and cultural barriers and enablers that have shaped the public perception and acceptance of Black women leaders.

By combining the speech specimens from the two repositories, we obtain a unique dataset of female leadership speech. Yet, there are several important aspects about the dataset, which should be noted and which we take into account when conducting our analysis. Initially, we have mined all specimen of text available from both repositories and obtained 3,207 specimens of text in total. The only mining criteria we apply is that the latest date of the text specimen should be December 31, 2019. We deliberately avoided collecting text specimens from 2020 and 2022 due to the prevalence of COVID-19-themed speeches in those two years. While analyzing data from 2020 and 2022 is of interest, it would be more appropriate to consider these data in a separate investigation. The female speeches in the text format were collected using scripts coded in Python 3.7.7. Of 757 women in our total database, 608 (80.3 percent) were American (see Supplementary Materials for raw data). Considering our concentration on the comparison of majority and minority leaders, we concentrated on 608 female leaders from the US. Apart from collecting specimens of leadership speech, the Iowa State repository also contains specimens of political advertisement. Political advertisements are samples of text representing transcripts of televised advertisements used by politicians as a part of their election campaign. These text specimens are not suitable for our analysis as they are produced for the purpose of winning the election, have the goal of attracting attention to a particular individual rather than describe this individual’s agenda, and often contain direct speech from other individuals. Therefore, we excluded all specimens of political advertisement obtained from the Iowa State repository and concentrated on speeches only.

For inclusion into each repository, the speech has to go through the review scrutiny by an editorial committee. As a result, there are a number of speeches which are selected for both repositories. We removed the duplicated speeches, which appeared in both repositories and obtained unique 2181 specimens of speech from 608 women leaders from the US. Speech data was added to the database.

Speeches hold a pivotal role in the realm of leadership^40^. They not only provide a platform for leaders to convey their vision, strategies, and goals but also help in building rapport with their audience. The form of a speech, encompassing its structure, language, and delivery, reflects the leader’s intent, preparedness, and approach to their subject. It becomes a mirror to the leader’s mindset, revealing nuances about their priorities, concerns, and aspirations. Functionally, speeches serve multiple purposes for leaders. They act as tools of motivation, education, and persuasion. Leaders use speeches to inspire teams, educate stakeholders about shifts in strategy or market dynamics, and persuade audiences to align with their viewpoint or vision. The content of a speech can influence public opinion, drive organizational change, or even reshape industry perspectives. Considering our dataset of female leadership speech, it is important to note that the speeches collated provide a rich tapestry of insights into the leadership styles, communication strategies, and priorities of the women leaders represented. This collection, while vast and diverse, serves as a unique reflection of the evolving dynamics of female leadership in the US, especially when observed through the lens of its form and function.

From the repository speech data, we obtained the name of individual, date of speech, place of speech, exact text of the speech, and occupation of the individual at the time of the speech, type of organization where an individual worked at the time of the speech. These data were also merged with publicly available information about each speaker. This information included race, nationality, as well as profession or professions. Considering that engaging into a new profession requires risk taking, the number of professions obtained and mastered by each female leader was taken as a proxy of their individual risk parameter. These publicly available data were mined from the Wikipedia pages as well as from the official website of each woman leader (where available).

**Figure 2:**
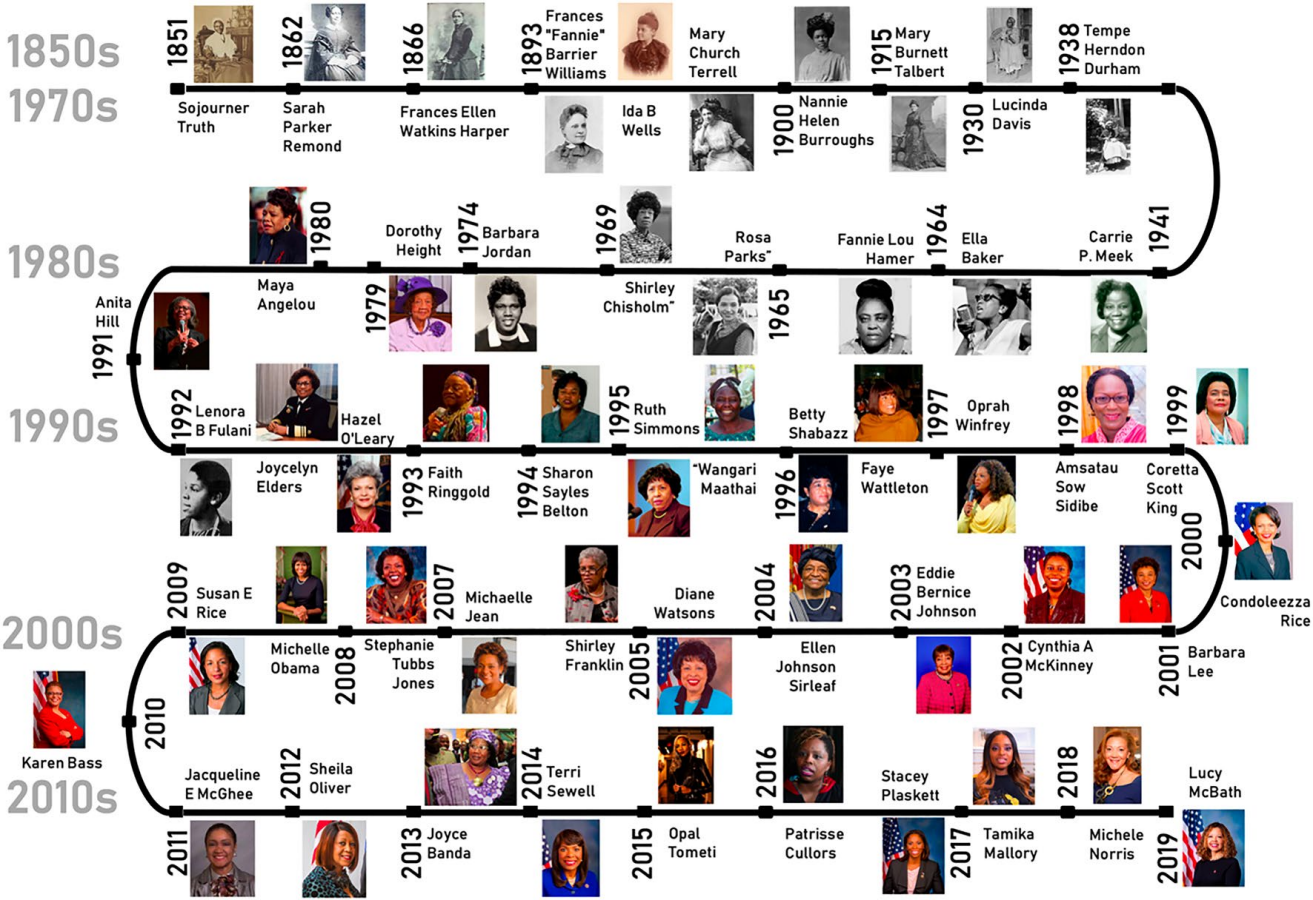
Selected Black Female Leaders’ Timeline. *Note* The figure shows at least one Black female leader per year. Where more than one woman leader was present, we have shown either all women if space permitted or selected one at random. If a female leader in our sample gave several speeches over a number of years, the year of the first speech in our sample was used for the figure.

Nationality information was available for all 608 women, only 4 of whom had dual nationalities (US plus one other country). In terms of ethnical and racial composition, of the 608 women in our sample, 462 identified themselves as White, 87—as Black or African American, 10-Asian, 18-Hispanic or Latino , and 10 had other single race background. In our sample, 21 women had either mixed background or unknown background. Specifically, we could not find background information about 10 women, and 11 women were identified having several racial or ethnical backgrounds simultaneously, which made it difficult to place them in any one group. Information about the racial background was compiled from three main sources, which were cross-checked to form a unified classification: (i) a women leader’s own words in speeches, (ii) Wikipedia pages, as well as (iii) relevant lists (e.g., list of African American politicians, etc.). Black or African American group represents the largest minority group in our sample (see Fig. 2).

Figure 2 shows minority female leaders in our database. Speeches from majority and minority leaders covered the period from 1828 to 2019. We obtained women leaders’ occupation information (main area of expertise), which was cross-checked using several sources, including the relevant speech repository pages about female leaders (Iowa State and Gifts of Speech repositories) and Wikipedia biography pages. In addition, we also collected data about all areas in which a particular female leader was a recognized expert (i.e., information about a particular leader’s profession). Hence, for each female leader in our database we had the main occupation and all areas where she was considered an expert. The speeches captured in the sample from this group spanned a period from 1851 to 2019 (i.e., pre-COVID19). As a result, we captured a diverse group of Black or African American women leaders, who represented a wide range of areas from activism and feminism to science and technology. Figure 2 showcases this heterogeneity.

Women in our database had different number of speech specimens per person. The average number of speeches per female leader was equal to 3.32 with the median of 1 and a standard deviation of 8.05. Over 70 percent of women in our sample had 1 speech specimen. There were 8 female leaders in the database with the number of specimens greater that 18: Carly Florina with 23 specimens, Madelaine Albright with 26 specimens, Elizabeth Dole with 27 specimens, Joni Ernst with 33 specimens, Carrie Chapman Catt with 45 specimens, Michelle Obama with 54 specimens, Elizabeth Warren with 64 specimens, and Hillary Rodham Clinton with 181 specimens. Importantly, many speeches from these female leaders were recorded in the same calendar year. For example, Hillary Clinton is the largest outlier in terms of number of speeches in our sample. Even though her speeches span a time period from 1969 to 2019, 64 of 181 of her speeches are from 2016 when she was running a presidential election campaign in the US. Similar pattern is observed for other outliers: for example, most speech specimens from Madeleine Albright (15 of 26) are from 1997 when she became the Secretary of State. In order to mitigate issues arising from multiple speeches per leader, our analysis takes into account the fact that some text specimens are from the same person (i.e., assumes that specimens from the same female leader are correlated). Furthermore, our text analysis is dynamic in the sense that it first considers the average trend in text from each individual in a particular year and only then calculates the overall behavioral schema trend for the whole year.

Our analysis is initiated with the utilization of a dataset comprising female leadership rhetoric for conducting a topic modelling exercise. Our corpus, designed specifically for this topic modelling, encompasses 2,181 speech samples attributed to 608 female leaders, collated between the years 1828 and 2019. This corpus includes over 3 million words. Following a pruning process that eliminates punctuation, frequently used function words, single occurrence words, and university names as well as organizational names, the total word count of the corpus stands at 1,000,401.

The final corpus contains both individual and temporal effects, necessitating consideration of the correlation of speech samples from the same leaders as well as the potential correlation of topics across time periods, with our unit of time being one year. Our approach addresses individual effects via the topic modelling process and temporal effects at a post-topic-modelling stage. This process is required since existing topic modelling methods are incapable of addressing both effects simultaneously. For instance, while Latent Dirichlet Allocation (LDA)-based Correlated Topic Model (CTM) procedures permit modelling of topic correlations, they are unable to model correlations between text samples from the same author. Similarly, Dynamic LDA permits the capture of topic evolution within a sequentially organised document corpus but cannot be jointly estimated with Correlated LDA due to its use of different distribution models.

## Results

Our findings can be categorized into three distinct areas. Initially, we use leadership rhetoric data to derive a suite of articulated behavioural schemas, expressed in the form of topics frequently discussed by female leaders. It is crucial to note that these speech samples originating from the same leaders demonstrate inherent correlations. During this initial phase, we deliberate whether the focus of these topics (i.e., behavioural schemas) is dependent on the leader’s racial heritage. More precisely, we investigate if there exists a unique specialization in the topics addressed by Black female leaders as contrasted with those addressed by White majority leaders. Subsequently, we delve into the evolution of behavioural schemas over time, unraveling the temporal dynamics of these schemas bifurcated by race. Finally, we amalgamate the insights gathered regarding behavioural schemas with those pertaining to female leaders’ risk-taking tendencies, with an aim to explore the differences in risk-taking patterns amongst leader populations of varying ethical origins.

### Behavioral schemas of female minority leaders

**Figure 3:**
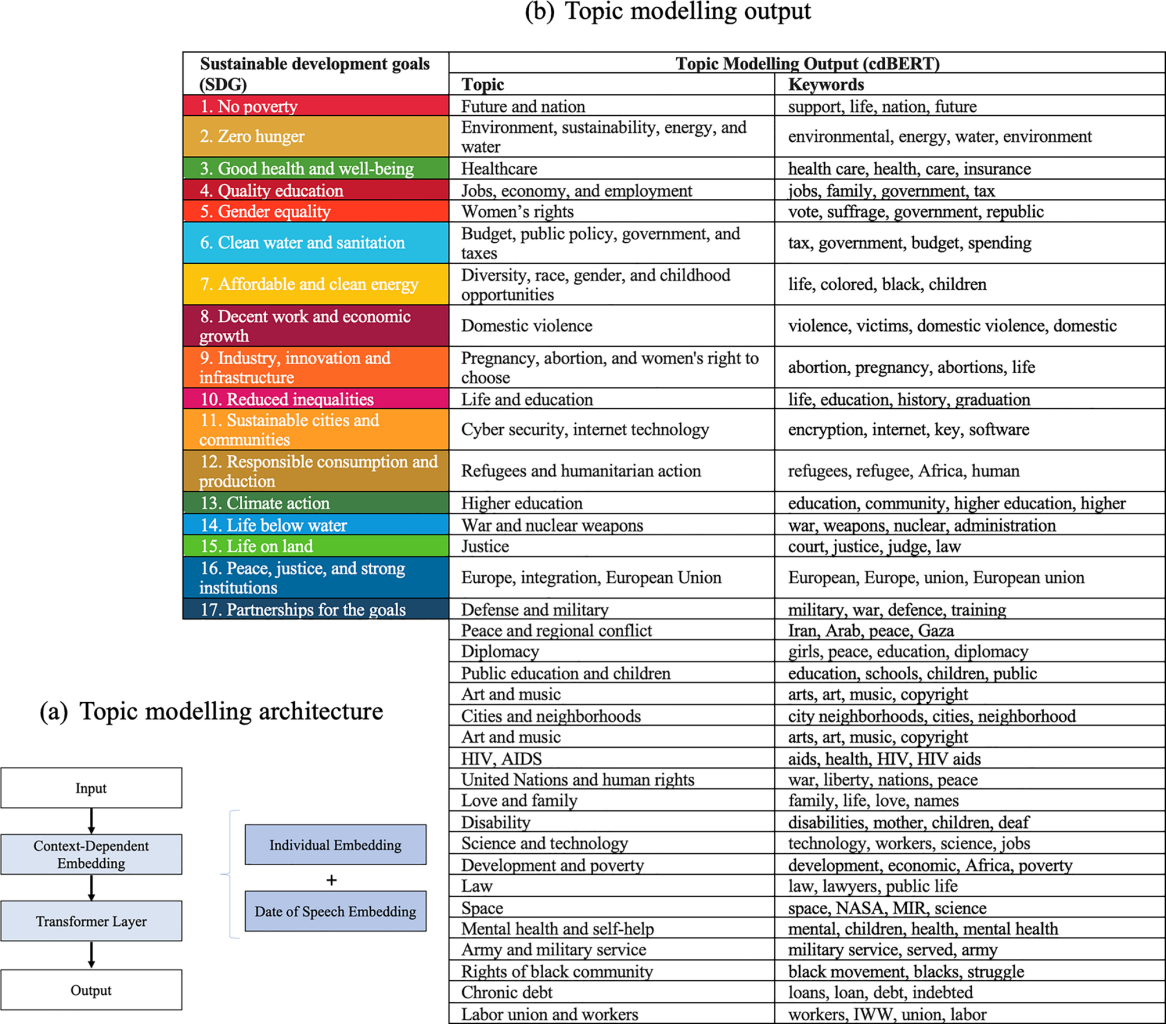
Architecture of Transformer Model and the Topic Modelling Output. *Note* The figure part (**a**) shows our topic modelling architecture, while part (**b**) demonstrates the topic modelling output.

Our approach first implements the topic modelling procedure accounting for individual-level dependencies, and subsequently measures individual and temporal effects using econometric methods in post-estimation. This methodology aligns with our focus on minority female leaders rather than a general temporal allocation of topics over time. To proceed with the topic modelling, we assume that each individual leader associates with a multinomial distribution of topics, and each topic associates with a multinomial distribution of words. A context-dependent Bidirectional Encoder Representations from Transformers (BERT)-based model (cdBERT) is employed. The method used in the cdBERT model is analogous to the approach used in contextualized models like Med-BERT by Rasmy et al. (2021), which utilise transformer architecture to integrate multi-level embeddings and bidirectional transformer (refer to Fig. 3).

In our application, individual leader ID and speech dates are used to differentiate between individual leaders and their respective speeches. Topic modelling is executed using Python 3.7.7, returning 35 topics. A detailed presentation of the topic modelling results can be found in Fig. 2 , which illustrates the alignment between topics within female leadership speech and the United Nations’ 17 global goals established in 2015.

**Figure 4:**
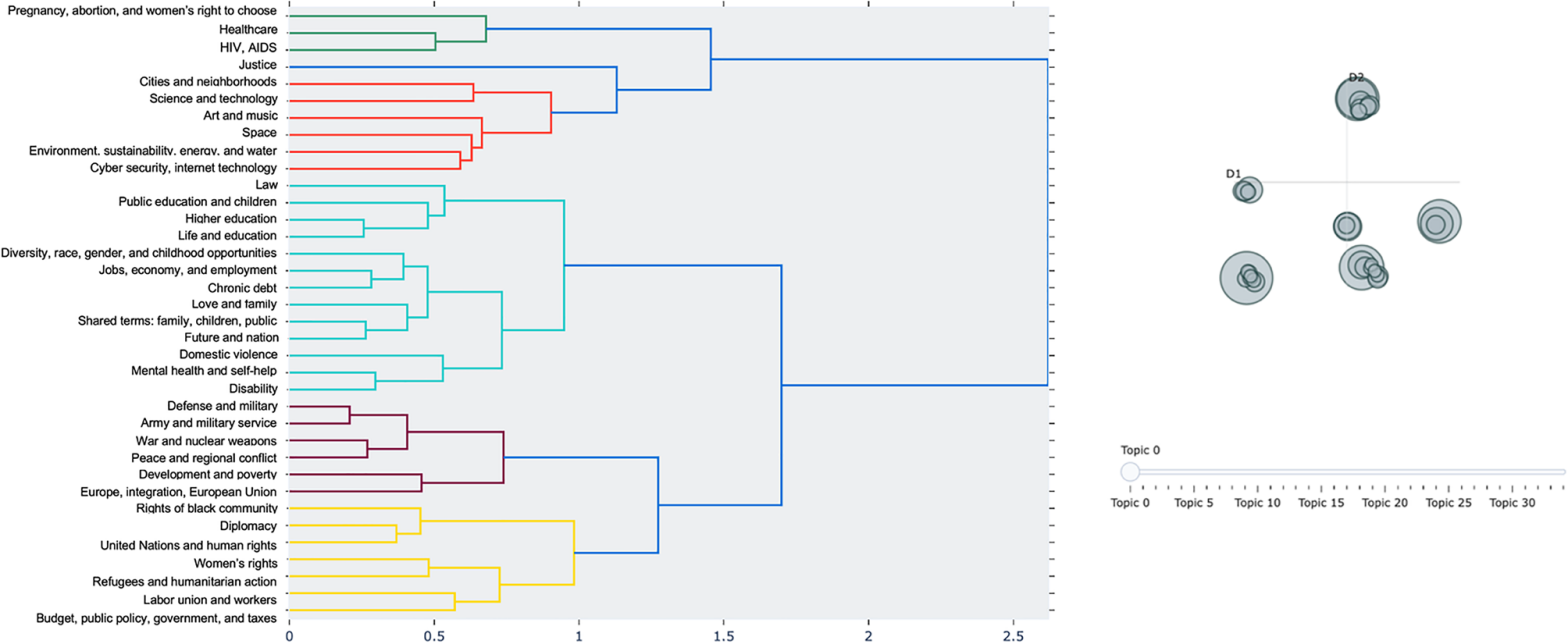
Hierarchical Clustering and Cluster Cross-distance Map. *Note* The figure demonstrates links and relationships between different topics in our analysis.

The BERT-based topics, although distinct, can be categorised into six groups via hierarchical clustering, as presented in Fig. 4. General behavioural schemas related to life, family, education, and economy form a distinct group, as do schemas related to war, peace, defence, and international politics. Another group focuses on equality, rights of underrepresented groups and minorities, and economic fairness.

**Figure 5:**
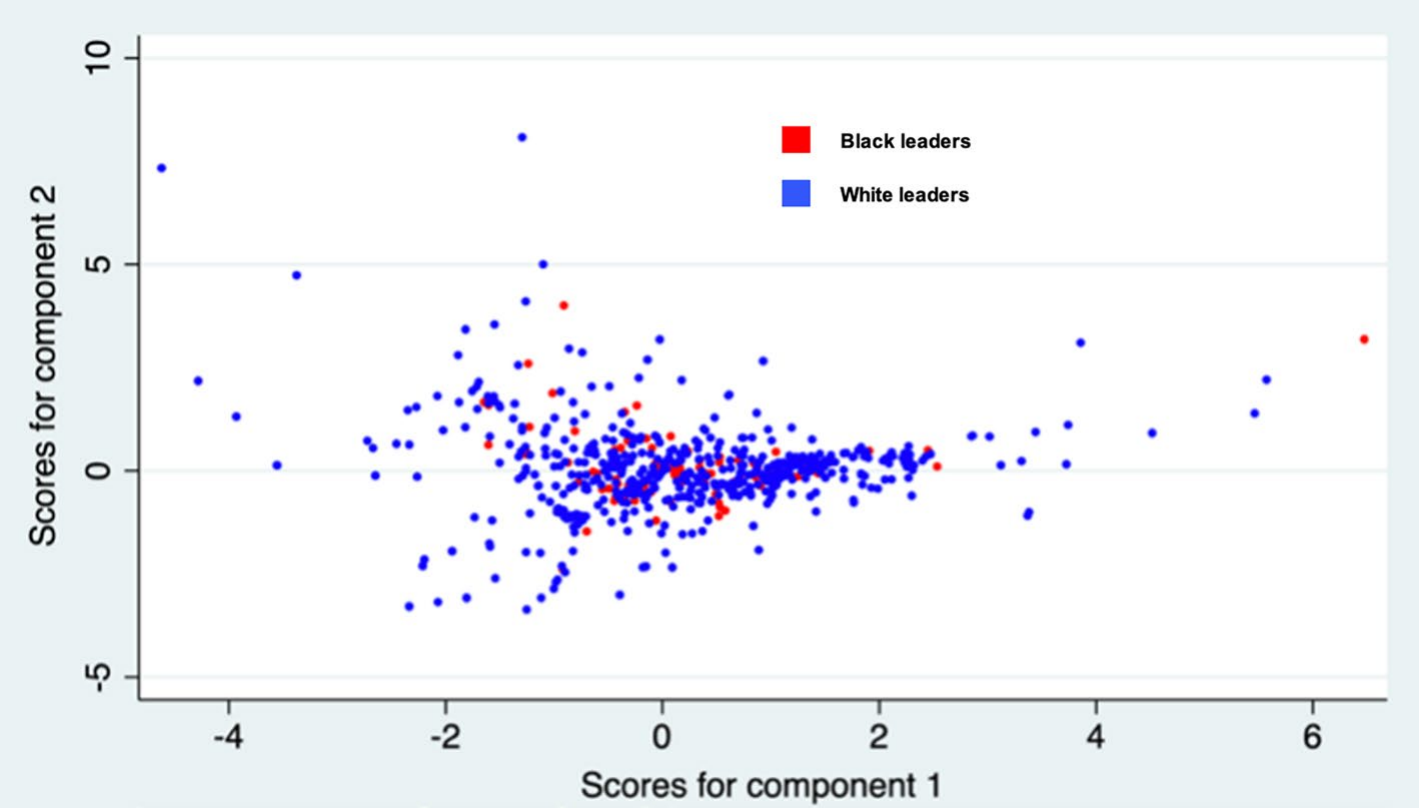
Two-dimensional Factor Mapping of the Relative Topic Distancing. *Note* Red dots represent Black (minority) female leaders and White (majority) leaders are captured by blue dots. Figure 5 displays the results of a factor analysis, where “Component 1” and “Component 2” are the first two principal components capturing the most significant underlying patterns in the data. These components, derived from the original 35 behavioural schemas identified through the topic modelling, simplify the topic modelling output complexity into a 2-dimensional graph.

A static analysis of the relative plot of White and Black female leader topic specialisations does not suggest clustering in our sample by ethnicity or racial background (see Fig.  5). Each dot represents an individual female leader, whose probabilistic 35-topic allocation (behavioral schema allocation) obtained from the topic modelling is dropped onto a 2-dimentional space. Female leaders with Black background are shown using the red color, while female leaders with any White background are depicted using blue color. Figure 5 shows no clustering of red and/or blue dots.

**Figure 6:**
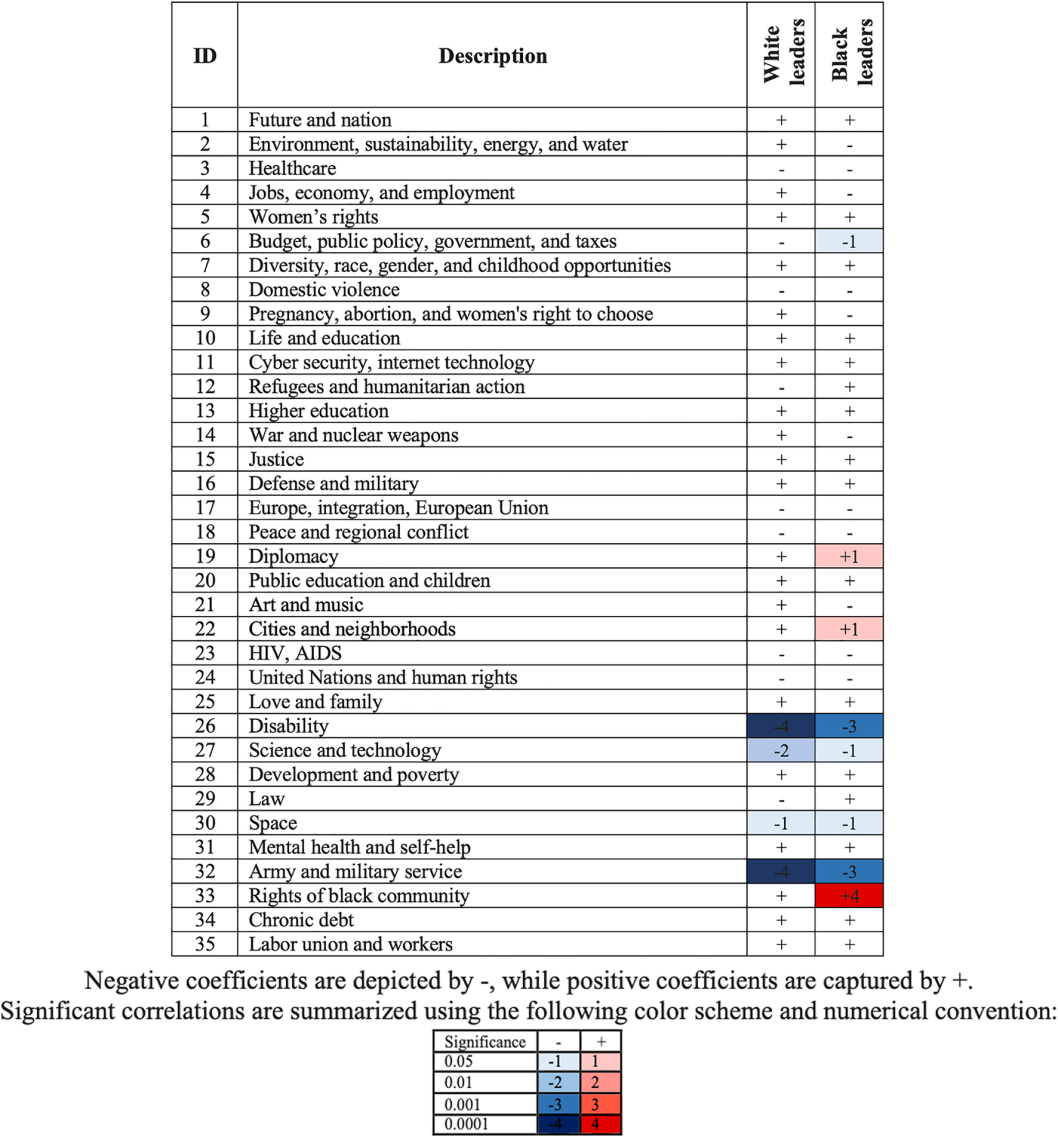
Results of A Series of Multilevel Regressions: A Summary.

We verify this outcome through econometric estimation and a series of multilevel regressions, measuring fixed effects at the level of each year at level 1 and each individual leader at level 2 (see Fig. 6).

Through the regression analysis, underrepresentation was identified in all female leader’s racial and ethnical groups in the behavioural schemas of Army and military service as well as Disability. A similar underrepresentation was observed in the Science and technology topic. Interestingly, Black leaders exhibited higher engagement in the Diplomacy topic and the areas of Rights of Black community as well as Cities and neighborhoods. All estimations control for the type of organization where a leader was employed at the time of the speech. Our analysis reveals no statistically significant differences between private, public, and third sector organizations. These observations suggest a degree of specialization for Black female leaders regarding racial discrimination and urban environments. Yet, it is apparent that static analysis does not allow to fully identify the differences between Black and White female leaders, which is why we turn to the dynamic analysis in the next subsection.

### Dispersion in the behavioral schemas

**Figure 7:**
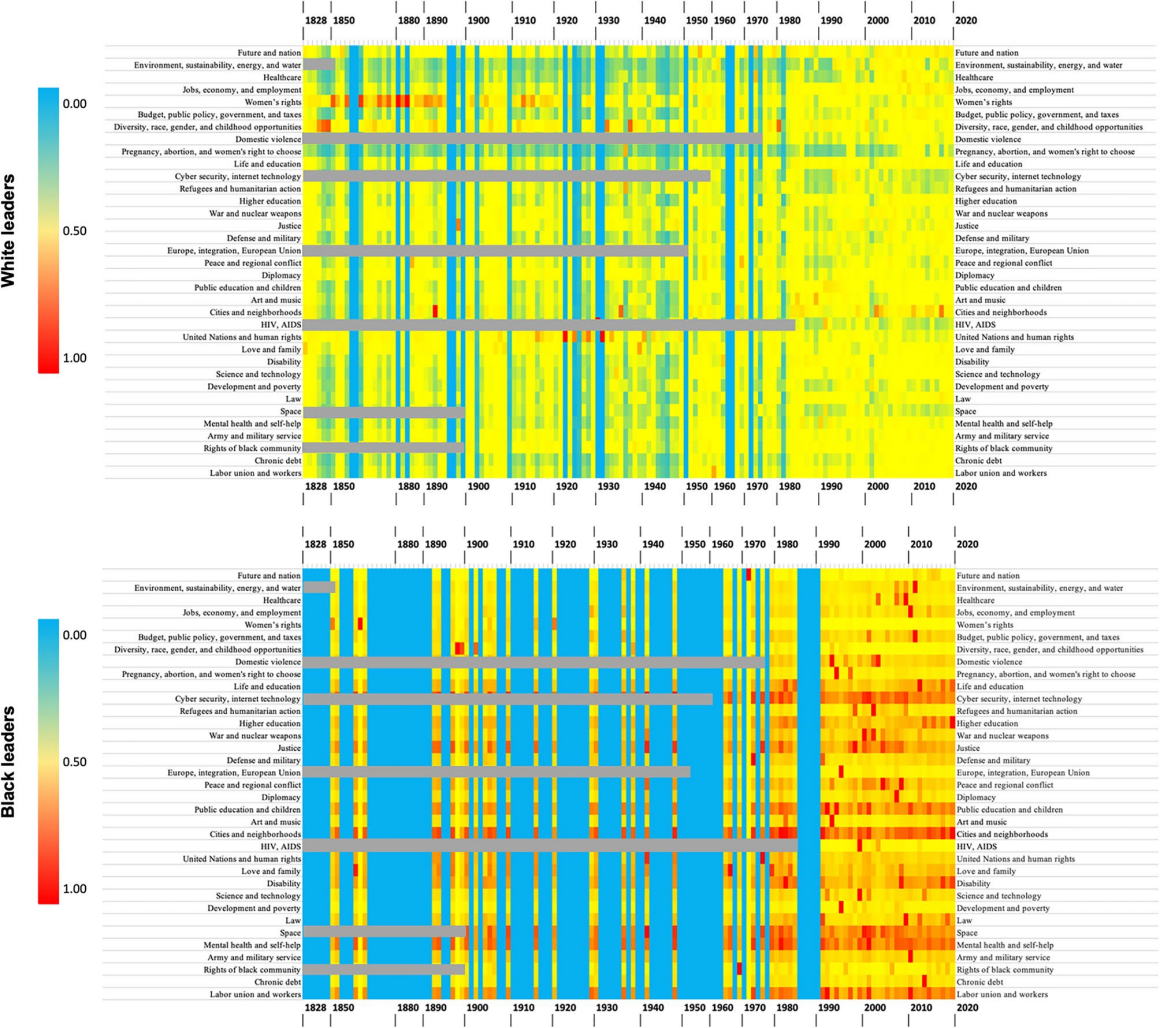
Temporal Evolution of Female Leaders’ Behavioural Schemas. *Note* Grey areas indicate the time periods when the identified topics were non-existent. For example, the term “Domestic violance” only appeared in 1973.

In this study, a temporal review of the behavioural schemas demonstrated by American female leaders spanning from the 19th to the 21st century was carried out. Interestingly, although these leaders share similarities in their areas of focus, a certain level of specialisation associated with race or ethnicity was identified. Consequently, a closer examination of the dynamic evolution of these behavioural schemas within each group was warranted. Hence, all 35 behavioural schemas were mapped across a temporal spectrum for each racial or ethnical group of female leaders. The outcomes of this mapping exercise are depicted in a heatmap (Fig. 7), which uncovers intriguing patterns. Cooler hues (such as blue) on the heatmap represent lower concentrations of a specific behavioural schema within the leadership rhetoric in a given year. Conversely, warmer colours (such as red) indicate a higher concentration.

Primarily, Figure 7 illustrates that, despite the propensity of majority leaders (those from a White background, labelled ’White’ in the figure) to frequently alter their behavioural schemas, the behavioural schemas of minority leaders (Black background, labelled ’Black’) exhibit greater temporal stability. Notably, the group of Black minority leaders consistently exhibit a strong focus on specific topics over time, including Justice, Public Education and Children, Cities and Neighbourhoods, Space, Cybersecurity and Internet Technology, Mental Health and Self-help, and Labor Unions and Workers.

Since our study utilizes a BERT-based algorithm for topic modeling of both contemporary and historical speeches, our deep learning model, trained on data, where modern data has much more significant representation than historical data, demonstrates its capability to contextually interpret and classify text into relevant topics. BERT’s design, which analyzes words in their full context rather than in isolation, enables a nuanced understanding of language. This approach is especially effective in identifying contemporary topics related to historical texts. For example, historical discussions about ’Space’ often encompass broader celestial elements, such as stars and the heavens, rather than modern conceptions of space travel or technology. Historical terms like ’telegraphy’, which date back to 1832, are linked to modern terms like ’Internet’; and terms like ’communications security’, known since World War II, are associated with contemporary terms like ’Cybersecurity’. To avoid confusion, grey areas in Figure 7 indicate years when the modern terms, identified by BERT as topics, did not exist. To determine the origin of a term, we first used an etymological dictionary, which provided detailed histories of words, including their first known usage. We then cross-checked this information with the Macroscope tool (http://macroscope.intelligence-media.com/), which offers a detailed historical analysis of terms, jointly with words associated with these terms.

Our findings imply that majority female leaders, who experience positive societal change over time, can adapt their focus swiftly, shifting their behavioural schemas to address new challenges as old ones are resolved. Meanwhile, minority female leaders, who do not experience such positive societal changes, maintain their focus on consistent behavioural schemas over time. Consequently, while rapid updates in the behavioural schemas of majority female leaders are observed, minority female leaders are compelled to maintain focus on consistent behavioural schemas. This conclusion holds true across private, public, and third-sector organisations.

**Figure 8:**
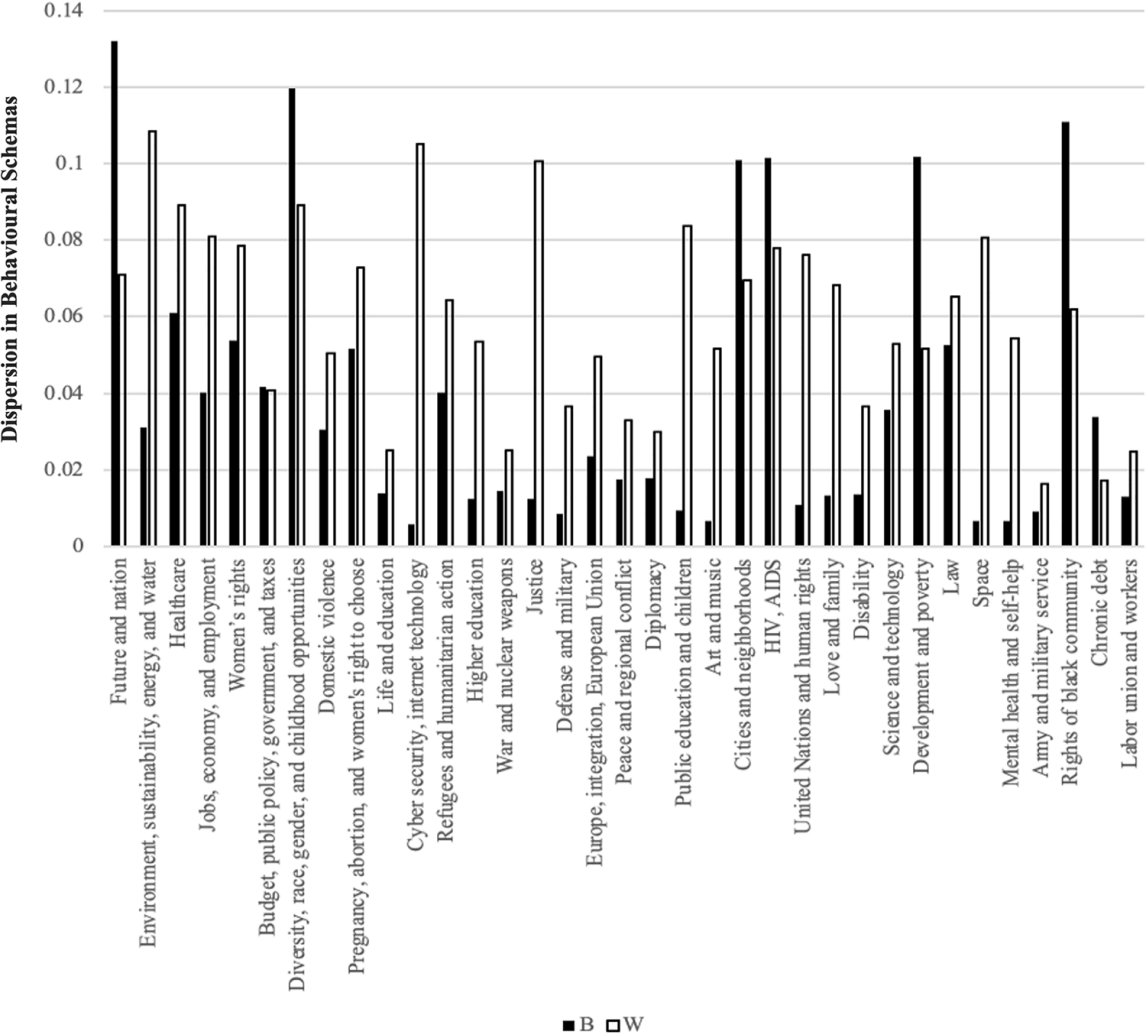
Comparison of Dispersion in the Behavioral Schemas between White (W) and Black (B) Leaders.

If our initial hypothesis (Hypothesis 1) is valid, not only should there be temporal invariance in the behavioural schemas of minority female leaders, but also a lower degree of dispersion in prevalence coefficients for the same behavioural schema (or topic) within the minority group compared to the majority group. To ascertain this, standard deviations of the prevalence coefficients for each topic were compared, with a specific focus on the contrast between White majority and Black minority female leaders. The outcomes of this comparison, presented in Fig. 8, reveal that 27 of 35 topics exhibit a greater dispersion in behavioural schemas in the majority group than in the minority group. The observed differences are statistically significant as per the F-test, thereby substantiating Hypothesis 1. This finding suggests that minority leaders have more time-invariant and less dispersed behavioural schemas than majority leaders, implying that they continuously grapple with the same issues over time, without observing progress in resolving these critical matters. Consequently, the rate at which behavioural schemas are updated for minority leaders lags behind that of majority leaders. This slower rate of update leads to a static behavioural schema for minority leaders over time, thereby influencing their psychological states, expectations, and professional development.

### Risk-taking

Our theoretical framework suggested that minority leaders, given their lack of progress over time, may resort to risk-taking as a form of compensation for this stagnation. This theory was backed by our analysis, which showed that minority leaders demonstrated a greater risk-taking propensity ($\sigma _i<\sigma _j$, thereby implying $r_i^t > r_j^t$).

We calculated the average number of areas entered and excelled in by each female leader in our study by collecting the data from the publicly accessible Wikipedia profiles. Taking Hazel R. O’Leary’s Wikipedia profile as an example, it states that “Hazel Reid O’Leary (born May 17, 1937) is an American lawyer, politician, and university administrator who served as the 7th United States secretary of energy from 1993 to 1997.” From this description, we identified and catalogued three distinct professions or areas: lawyer, politician, and university administrator/educator. To ensure the precision of our data extraction, manual cross-referencing was undertaken using individual official websites, such as Hazel O’Leary’s website. For Hazel R. O’Leary, based on our methodology, the risk parameter was established at a value of 3, representing the three professional areas she engaged in and excelled at. This systematic approach was employed for all female leaders incorporated in our study. While White female leaders on average engaged in 1.59 areas, Black female leaders ventured into an average of 2.13 fields, indicating a significantly higher risk-taking propensity. This was further corroborated by the Mann-Whitney-Wilcoxon test, which validated these differences as statistically significant (z = − 5.708,* p* = 0.0000).

These findings confirm our Hypothesis 2: minority female leaders, in the face of a lack of positive societal changes and behavioral schema updating, compensate for this stagnation by demonstrating higher risk-taking behavior.

The analysis of longitudinal data further solidified our findings, showing a diminishing risk-taking trend among White female leaders over time (estimation of trend function revealed a declining pattern with the coefficient of − 0.0054). In contrast, Black female leaders displayed an increasing propensity towards risk-taking (estimation of trend function revealed a declining pattern with the coefficient of 0.0084). This pattern not only confirms our Hypothesis 3, but also provides additional insights into the dynamic landscape of risk-taking behavior across racial and ethnic groups in female leadership.

These results provide a comprehensive, nuanced understanding of the behavioral schemas of female leaders across racial and ethnic groups and over time. Our findings underscore the importance of recognizing the distinct experiences and challenges faced by minority female leaders. They call attention to the need for devising policies and strategies that are not only supportive but also empowering, tailored to the unique needs and experiences of minority female leaders.

## Discussion

Many studies talk about racial inequality in organizational leadership identifying important problems faced by the minority female leaders (see, e.g., Parker 1996 for early literature on Black female leadership). Yet, to date, few attempts were made to quantify racial bias and measure its impact on women leaders. In this paper, we propose a new theoretical behavioural data science approach inspired by the Bourdieusian leadership framework^19^ and building on the dynamic leadership development models by^20^ as well as^21^ and test this framework quantitatively using a large sample of female speech.

Recent literature highlights the significant underrepresentation and challenges faced by Black female leaders in the corporate sector^41^. According to the 2020 report by LeanIn, for every 100 men promoted to their first managerial role, only 58 Black women receive a similar advancement. One in 3 Black women feel they are less empowered and supported to overcome professional challenges than the general population. Additionally, the IBM Institute for Business Value (IBV) found that over half of Black women perceive discrimination against them in their workplace based on race. Furthermore, while 84% of Black women believe there is discrimination against women, only 64% of White women share this sentiment. Despite these challenges, Black women exhibit a strong drive for leadership; research from the Center for Talent Innovation notes that they are more likely than White women to aspire for executive leadership roles. This underlines the importance of organizations to recognize and address the unique hurdles Black female leaders face.

Using 200 years of female leaders’ rhetoric history in the US, we demonstrate that even though a static view shows that women representing different backgrounds are not different in their aspirations to make the world a better place, dynamic analysis reveals that sustained intersectional effects (due to a combination of gender and racial bias) produce unequal outcomes for minority and majority leaders. This study demonstrates that Black female leaders often take on greater risks to address the compounded challenges of intersectional invisibility. Yet, compared to their White peers, their goals often remain unachieved. This has resulted in recurring challenges that successive generations of Black female leaders have had to confront for extended periods, spanning decades and even centuries.

Specifically, female leaders representing racial minorities tend to experience lack of change in their social outcomes over time due to sustained discrimination. This causes the lack of updating in their behavioral schemas—i.e., their perceptions of the world and their place in the world. As a result, for many generations, they tend to focus on the same problems, which fail to get resolution over time. This lack of experiential change and time-invariant behavioral schemas cause them to engage in more and more risky behaviors in order to succeed. Using the Burna Boy’s (an award winning rap artist’s) analogy, we find that Black female leaders have to be “twice as tall” compared to their majority counterparts in order to succeed, suffering from significant adverse effects of racial bias.

In examining the challenges encountered by female leaders from racial minority backgrounds, it is imperative to elucidate the profound implications of Burna Boy’s “twice as tall” metaphor. This analogy, while resonant, is emblematic of the broader principle of “working twice as hard” to attain equivalent success within an inherently biased system. This sentiment has its roots in the socio-medical construct of John Henryism^42^, which delineates the exhaustive endeavors of Black individuals, often culminating in adverse health outcomes. Concurrently, the theory of Racial Battle Fatigue highlights the psychological duress experienced by individuals of color due to incessant racial microaggressions. Together, these frameworks underscore the profound physiological and psychological toll exacted by the persistent drive to surpass racialized expectations. In any discourse regarding the experiences of Black female leaders, it is paramount to acknowledge these multidimensional challenges, born from a legacy of systemic discrimination.

Even though we observe some degree of specialization, similar to the studies highlighting the inconsequentiality of the socially constructed notions of race, our analysis of Black female leadership reveals that race has not determined the priorities and interests of global female leaders. Yet, understanding of female minority leadership in organizations has been incomplete as in many societies we continue to consider static rather than dynamic effects of intersectionality. Much of the organizational diversity training if focused on acceptance and equality at a particular moment in time rather than through time. Yet, our study shows that there are rigid behavioral schemas and negative experiences, which affect minority leaders from generation to generation, making them take more and more risk in order to fight the lack of positive experiences as well as intersectional invisibility.

This suggests that diversity in leadership should be addressed by organizations holistically as a dynamic concept, concentrating around improving the outcomes not only for one particular leader or a particular group of (minority) leaders, but rather increasing the number of positive experiences for the entire generation of leaders and promoting positive change. Our findings demonstrate that it is not enough to address diversity through increasing the number of minority leaders by creating promotion opportunities. Rather, it is important to facilitate the change in their behavioral schemas through positive experiences such that more minority leaders can emerge. Currently, becoming a successful leader has a very costly barrier for minority leaders—they face a large cost to entering the leadership scene, because they are required to take a lot of risk to succeed. If they start to see that problems are resolved and if their experiences become more positive, minority representatives will enter the leadership scene more as the cost of entry will be reduced because they will not need to compensate the lack of change with risk taking.

The core argument in many previous studies has revolved around identifying the issues faced by minority female leaders, primarily through qualitative means or non-intersectional quantitative analyses^12^. While these studies have been instrumental in shedding light on racial inequality in leadership, our research takes a pioneering step by quantitatively merging the concepts of intersectionality with leadership rhetoric spanning 200 years in the US. The emphasis on intersectionality, drawing from both gender and racial perspectives, allows us to observe unique trends and outcomes that would otherwise remain unseen in a non-intersectional approach. In a non-intersectional quantitative study, these challenges might be reduced to singular issues related to either gender or race. Similarly, qualitative studies, while rich in detail, may not always be able to highlight the recurrent patterns and sustained effects over extended periods as a quantitative approach can. By juxtaposing the static and dynamic effects of intersectionality, our study reveals that while aspirations of women leaders across various backgrounds remain consistently benevolent, their outcomes and experiences significantly diverge due to the intersection of gender and racial bias.

It is paramount to emphasize that the success of diversity initiatives should not be solely measured by increasing the representation of minority leaders. Instead, a more holistic approach, as suggested by our findings, should consider improving the experiential landscape for these leaders, addressing generational challenges, and facilitating changes in their behavioral schemas through positive experiences. In essence, this research bridges a critical gap by providing a quantitative testament to the intersectional challenges minority female leaders face, emphasizing the need for systemic and long-term solutions in organizational settings.

It is also important to note that black women have historically navigated a landscape where their speech and actions were heavily restricted. Our results suggest that this constraint was entrenched in systemic racial and gender biases, limiting their opportunities to assert leadership and influence. The historical narrative for white women, while also marked by gender-based restrictions, presented a relatively broader scope for self-expression and leadership, albeit within confined boundaries. This differential historical experience has invariably shaped the trajectory of leadership behavior and opportunities for women of different racial backgrounds.

These historical constraints may have contemporary implications. For black women, the enduring legacy of more stringent speech restrictions has likely translated into a form of leadership that often necessitates cautious navigation of both racial and gender norms. In contrast, white women’s leadership behaviors, though still influenced by gender norms, have evolved differently due to their historical access to relatively more freedom of speech and action.

By integrating these historical nuances into our analysis, we deepen our understanding of how intersectional factors shape leadership behaviors. It is crucial to acknowledge that the leadership landscape for women is not monolithic; it is sculpted by the complex interplay of race, gender, and historical context. This recognition allows us to more accurately capture the varying experiences and challenges faced by black and white women in leadership roles. We propose that future research should further explore how these historical constraints continue to influence contemporary leadership styles and opportunities for women of different racial backgrounds. Such research could provide valuable insights into the development of more inclusive and effective leadership strategies that recognize and address the unique challenges faced by women of diverse racial and gender identities.

Our study has a number of limitations, which could be addressed by the future research. Our model assumes that the risk-taking decision and leaders’ experiences are separable. This limitation can be addressed by considering a Bayesian modelling approach, where a leader starts with a set of priors and then updates her priors with each experience. We also assume that gender and ethnicity identification of a leader does not change over time. Our model allows to relax this assumption by introducing endogenous or exogenous shocks to the priors and measuring how the changes in priors affect outcomes. Finally, we capture individual effects of leaders by econometric and statistical means (e.g., via fixed effects). Another option would be to collect more detailed (preferably experimental) data on leaders measuring their behavioral traits, risk attitudes, as well as other individual variables in order to explore the link between these characteristics and leadership rhetoric. It is left to future research to explore these exciting endeavors, which would allow to expand our understanding of dynamic and intersectional effects of racial and gender bias and their impact on minority female leadership in organizations.

### Supplementary Information

Supplementary Information.

## Data Availability

To simplify the refereeing process, the data, analysed in this paper, is provided in the Supplementary Materials to this manuscript.
