# Supplementary material for: The impact of intersectional racial and gender biases on minority female leadership over two centuries
Source: Sci Rep. 2024 Jan 2;14:111. doi: 10.1038/s41598-023-50392-x (PMC10762112; doi:10.1038/s41598-023-50392-x)
Supplement: Supplementary file 1 — Supplementary Information. [file 41598_2023_50392_MOESM1_ESM.pdf]

Supplementary Information

(NOT INTENDED FOR PUBLICATION)

Appendix A

Example of Excluded Political Advertisement

We exclude speech specimens containing deliberate political advertisement, which are much shorter than those in our dataset and have a particular structure, usually heavily regulated (e.g., the candidate always must endorse the message by stating they "approve" it). Such political advertisements are usually not informative as their main goal is to attract attention to the candidate to encourage voters to look for more information about the candidate rather than to state the candidate's point of view. Such political advertisement often also include content from other people. We present two examples of political advertisement for Hillary Clinton below:

Example 1:

She Always - Sep. 20, 2016

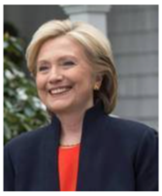
Hillary Rodham Clinton

Hillary Rodham Clinton
September 20, 2016
Print friendly
Video
Campaign status: Lost
CATEGORIES: POLITICAL ADS, TV

CLINTON: I've always been familiar with poverty, and I've always done what I could to alleviate it and to help people. The rich are getting a whole lot richer, the middle is stagnating, and the poor are getting poorer. Now that is not good news for the American democracy. This country needs to give dignity to work, to make sure that women and men who work full-time earn a wage that lifts them out of poverty. Corporate executives have profited personally so much when the average worker in America have seen their wages and benefits basically stagnate. A new beginning that makes Wall Street shoulder its responsibility for this crisis. One that makes the most well-off among us pay our fair share. We all want an economy with more opportunity and less inequality. Where Wall Street can never wreck Main Street again. We need to make sure our economy works for everyone, not just those at the top. I'm Hillary Clinton, and I've always approved this message.

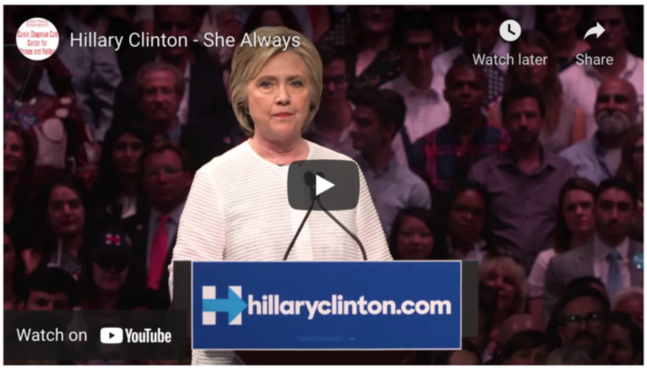

Example 2:

First Lady Michelle Obama on Voting - Nov. 3, 2016

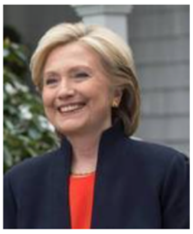
Hillary Rodham Clinton

Hillary Rodham Clinton
November 03, 2016
Print friendly
Video
Campaign status: Lost
CATEGORIES: POLITICAL ADS, WEB EXCLUSIVE

MICHELE OBAMA: Voting is how we express out values and choose the leaders we trust to shape the future for our children. And in this election, I'm casting my vote early for Hillary Clinton, and I urge you to vote early for Hillary, too.

MAN: You know that your vote counts. It's very important that we all get out and vote.

MICHELE OBAMA: I'm voting for Hillary because she shares my values, because she believes in equality, opportunity, hard work. I'm voting for Hillary because after her career as a lawyer, a law professor, First Lady of Arkansas, First Lady of the United States, a U.S. Senator, and Secretary of State, Hillary has more experience and exposure to the Presidency than anyone in our lifetime. And she is by far the most qualified candidate in this election. And I'm voting for Hillary because she has concrete, detailed policies that will actually make a difference in people's lives. Making college affordable, helping families pay their bills, improving our children's schools, and so much more. So Hillary has done her job, now it's time for us to do our job.

MAN #2: She's been a champion for us, and I want to be a champion for her.

MICHELE OBAMA: And get her elected President of the United States.

WOMAN: We're electing the first female president!

WOMAN #2: I'm so excited!

MICHELE OBAMA: That's why folks across this country, including Barack and I, are voting early. And early voting couldn't be easier. Barack voted early in person by showing up to an early vote location in our hometown of Chicago.

BARACK OBAMA: Ok, let's do this.

MICHELE OBAMA: So vote early if you can or you can join the millions of Americans who will head to the polls on Election Day, November 8. However you choose to cast your vote, just be sure that you make your voice heard and help elect Hillary as our next president. Thanks so much!

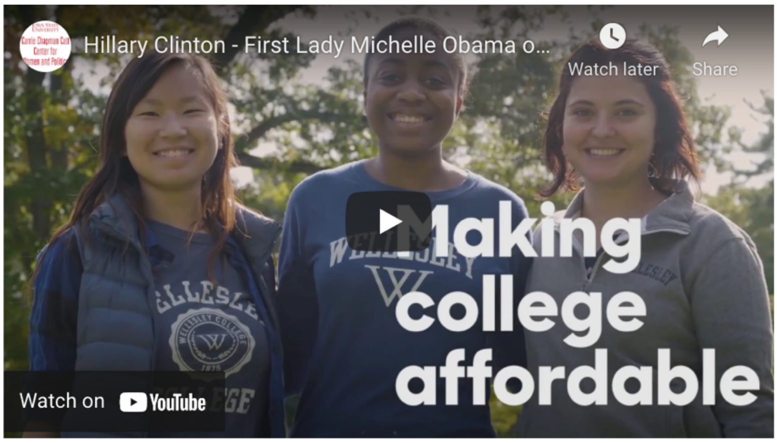

Appendix B

Table B1 List of Female Leaders in the Originally Mined Dataset (includes all US and non-US leaders)

Abby Finkenauer	Aung San Suu Kyi	Carmen Perez	Cristina Fernandez de Kirchner
Abby Kelley Foster	Aurelia E. Brazeal	Carol Bellamy	Crystal Eastman
Ada Deer	Ayanna Pressley	Carol Browner	Cynthia A McKinney
Adelle Hazlett	Ayoola Obe	Carol Molnau	Cynthia M Lummis
Alexandria Ocasio-Cortez	Barbara A. Goodno	Carol Moseley Braun	Cynthia M. Patterson
Alexandrina Rusu	Barbara B Kennelly	Carol Shea-Porter	Daisy Bates
Alexis Herman	Barbara Bush	Caroline Kennedy	Dale S. Brown
Alice M. Rivlin	Barbara Comstock	Carolyn B Maloney	Dale Spender
Alice Paul	Barbara Coombs Lee	Carolyn McCarthy	Dalia Grybauskaite
Alicia Garza	Barbara Cubin	Carrie Chapman Catt	Dana Loesch
Alison Lundergan Grimes	Barbara Dooley	Carrie P. Meek	Darlene K Hooley
Aliza Sherman	Barbara Ehrenreich	Catherine Bertini	Deb Fischer
Allee Willis	Barbara Jordan	Catherine Cortez Masto	Debbie A Stabenow
Allyson Schwartz	Barbara L Boxer	Catherine Jay Didion	Debbie Dingell
Alma Adams	Barbara Lawton	Catherine, Duchess of Cambridge	Debbie Halvorson
Alva Myrdal	Barbara Lee	Cathy Cleaver	Deborah Blum
Amata Coleman Radewagen	Barbara McClintock	Cathy McMorris Rodgers	Deborah Morris
Amelia Earhart	Barbara Mikulski	Cecile Richards	Deborah Y. Parker
Amsatou Sow Sidibe	Barbara O'Brien	Chandrika Bandaranaike Kumaratunga	Debra Stewart
Amy Kaslow	Barbara P Bush	Charlene Barshefsky	Denise Levertov
Amy Klobuchar	Barbara S. Cochran	Charlotte B Nelson	Diana Aviv
Amy Kremer	Becky Cain	Charlotte Bunch	Diana DeGette
Amy McGrath	Becky S Skillman	Charlotte van Rappard-Boon	Diana Princess of Wales
Andrea Dworkin	Begum Khaleda Zia	Chellie M Pingree	Diane Black
Andrea L. Ireland	Bella Abzug	Chelsea Clinton	Diane Denish
Angela D Merkel	Benazir Bhutto	Cheri Bustos	Diane Nash
Angelina Grimke Weld	Bernadine Healy	Cherie Blair	Diane P. Wood
Anita Borg	Bertha von Suttner	Christiane Amanpour	Diane Watson
Anita Hill	Bethany Hall-Long	Christiane Nuesslein-Volhard	Dianne Feinstein
Anita Jones	Betty A Ford	Christina D Romer	Dilma Rousseff
Anita Roddick	Betty Friedan	Christine Gregoire	Dina Titus
Ann Coulter	Betty McCollum	Christine Lagarde	Dolores Huerta
Ann Kirkpatrick	Betty S Sutton	Christine Todd Whitman	Donna Christensen
Ann M. Veneman	Betty Shabazz	Cindy A. Cohn	Donna Edwards
Ann Marie Buerkle	Betty Williams	Cindy Axne	Donna Shalala
Ann McLane Kuster	Beverly E Perdue	Cindy H McCain	Doris Kearns Goodwin
Ann Richards	Beverly O'Neill	Claire McCaskill	Doris Matsui
Ann Wagner	Billie Jean King	Claire Wolfe	Dorothy Crowfoot Hodgkin
Anna Eshoo	Blanche Lambert Lincoln	Clare Boothe Luce	Dorothy Denning
Anna Howard Shaw	Bonnie Kestner	Clare Short	Dorothy Height
Anna Kelton Wiley	Bonnie Watson Coleman	Claudia Alta "Lady Bird" Johnson	Dr. Kathleen C. Bailey
Anna Lindh	Brenda Laurel	Claudia Tenney	Dr. Rita Colwell
Anne C. Petersen	Brenda Lawrence	Cokie Roberts	Eddie Bernice Johnson
Anne K. Bingaman	Bronwyn Bishop	Colleen Hanabusa	Eileen B. Claussen
Anne M Mulcahy	Brooke Astor	Condoleezza Rice	Eileen Collins
Annie Sprinkle	Callie Khouri	Constance A. Morella	Eilene Galloway
Antonia Novello	Camille Paglia	Constance Yai	Elaine L Chao
Arundhati Roy	Candice Miller	Corazon Aquino	Elaine Scruggs
Atifete Jahjaga	Candy Crowley	Coretta Scott King	Eleanor Baum
Audrey McLaughlin	Carly Fiorina	Courtney Love	Eleanor Holmes Norton

Table B1 continued

Eleanor Roosevelt	Frances Moore Lappe	Ingrid Newkirk	Jill Biden
Eleanor Smeal	Frances Perkins	Irene Joliot-Curie	Jo Ann Harris
Elena Kagan	Frances Willard	Isabella Beecher Hooker	Jo Jorgensen
Elisabeth Showalter Muhlenfeld	Frances Wright	Ivanka Trump	Joan Brown Campbell
Elise Stefanik	Frederica Wilson	Iveta Radicova	Joan Claybrook
Elizabeth "Betsy" DeVos	Gabrielle Giffords	J.K. Rowling	Joan Ruddock
Elizabeth "Liz" Cheney	Gale Norton	Jacinda Ardern	Jody Williams
Elizabeth Cady Stanton	Geraldine A Ferraro	Jackie Speier	Joni Ernst
Elizabeth Dole	Gertrude B. Elion	Jackie Walorski	Josephine M. D'Antonio
Elizabeth Dowdeswell	Geun-hye Park	Jacklyn "Jacky" Rosen	Joy Hakim
Elizabeth Esty	Gill Marcus	Jacqueline E McGhee	Joyce Banda
Elizabeth Glaser	Gina Raimondo	Jacquelynn Davis	Joyce Beatty
Elizabeth Gurley Flynn	Ginette Hemley	Jadranka Kosor	Joyce C. Lashof
Elizabeth H Roberts	Ginny Brown-Waite	Jaime Herrera Beutler	Joycelyn Elders
Elizabeth McAlister	Gloria Macapagal-Arroyo	Jamie Gorelick	Juanita Millender-McDonald
Elizabeth Warren	Gloria Shatto	Jamie Rappaport Clark	Judith A. Best
Ella Baker	Gloria Steinem	Jan Brewer	Judith Crist
Ella T. Grasso	Golda Meir	Jan Schakowsky	Judith E. Heumann
Ellen DeGeneres	Grace Meng	Jane Addams	Judith L. Palkovitz
Ellen Greenlee	Grace Napolitano	Jane Alexander	Judith Lichtman
Ellen Hancock	Greta Thunberg	Jane Butler Kahle	Judith Rich Harris
Ellen Johnson Sirleaf	Gretchen Driskell	Jane Dee Hull	Judith Rodin
Ellen S. Hurwitz	Gretchen Whitmer	Jane Fonda	Judith Wallerstein
Ellen Tauscher	Gro Harlem Brundtland	Jane Harman	Judy Biggert
Elousie Cobell	Gwen Graham	Janet A Napolitano	Judy Chu
Emily Cain	Gwen Ifill	Janet G. Bostwick	Julia Brownley
Emily Greene Balch	Gwen Moore	Janet Jagan	Julia Gillard
Emma Goldman	Haley Stevens	Janet Mills	Julia Taft
Emma Gonzalez	Hanan Al-Shaykh	Janet Reno	Julia Ward Howe
Emma Watson	Hattie Caraway	Janet Southby	Kamala Harris
Emmeline Pankhurst	Hazel O'Leary	Janet Yellen	Kamla Persad-Bissessar
Erica Jong	Heidi Heitkamp	Janice Hahn	Karen Bass
Erna Solberg	Helen Alvare	Janice Shaw Crouse	Karen Hughes
Ernestine L. Rose	Helen Boosalis	Jari Askins	Karen M. Torres
Ester Dyson	Helen Chenoweth	Jean A. Wright	Karen Pence
Eva Clayton	Helen E Clark	Jean M Schmidt	Karyn Polito
Eva Persson Goransson	Helen Gahagan Douglas	Jeane J Kirkpatrick	Kate Michelman
Eveline Widmer-Schlumpf	Helen Gurley Brown	Jeanne Diebolt	Katha Pollitt
Evelyn Sanquinetti	Helen Hamilton Gardener	Jeanne Hurley Simon	Katharine T. Bartlett
Faith Ringgold	Helen Keller	Jeanne Shaheen	Katherine "Kate" Brown
Fannie Lou Hamer	Helen Prejean	Jenean Hampton	Katherine Clark
Faye Wattleton	Helle Thorning-Schmidt	Jennifer Carroll	Katherine Lund Hicks
Florence J. Harriman	Mrs Henry Weddington	Jennifer Dunn	Katherine O'Regan
Florence Kelley	Herta Muller	Jennifer Long	Katherine P. Prescott
Fran Ulmer	Hilda Solis	Jennifer M Granholm	Kathie Lee Gifford
France Anne Cordova	Hillary Rodham Clinton	Jenniffer Gonzalez	Kathleen B Blanco
Frances "Fannie" Barrier Williams	Ida B Wells	Jenny Beth Martin	Kathleen Kennedy Townsend
Frances "Sissy" Farenthold	Ida H. Harper	Jenny Horne	Kathleen Rice
Frances D. Gage	Ilhan Omar	Jenny Shipley	Kathleen Sebelius
Frances Ellen Watkins Harper	Indira Gandhi	Jessica Gonzalez-Rojas	Kathleen Sullivan

Table B1 continued

Kathryn Fuller	Lois Frankel	Marine Le Pen	Michele Reagan
Kathy A Dahlkemper	Lois J. Schiffer	Marion C. Blakey	Micheline Calmy-Rey
Kathy Castor	Loretta Lynch	Marjorie Dannenfelser	Michelle Bachelet
Kathy Hochul	Loretta Sanchez	Marsha Blackburn	Michelle Lujan Grisham
Katie Louchheim	Louise Arbour	Marsha Rosenbaum	Michelle Nowlin
Katie McGinty	Louise Frechette	Martha Krebs	Michelle Nunn
Katrin Jakobsdottir	Louise M Slaughter	Martha McSally	Michelle Obama
Katrina Pierson	Lucille Roybal-Allard	Martha Roby	Mimi Walters
Kay Bailey Hutchison	Lucinda Davis	Mary A. Safford	Molly Corbett Broad
Kay Granger	Lucretia Mott	Mary Anderson	Molly Ivins
Kay Hagan	Lucy McBath	Mary Bono	Monica Wehby
Kay Ivey	Lucy Stone	Mary Brown Bullock	Morgan Carroll
Kazimira Prunskiene	Luisa D Diogo	Mary Burke	Mother Teresa
Keli Carender	Lupe Valdez	Mary Burnett Talbert	Mrs. Asha Das
Kelli Ward	Lyn Stinson	Mary Carter Smith	Mrs. Hak Ja Han Moon
Kelly Ayotte	Lynn Jenkins	Mary Cavanaugh	Mrs. Sia Koroma
Kelly H. Carnes	Lynn Sherr	Mary Church Terrell	Mrs. T. J. Blankert-van Veen
Kellyanne Conway	Lynn Woolsey	Mary D. Nichols	Ms. Holmes
Kersti Kaljulaid	Lynne Cheney	Mary E Kramer	Muriel Bowser
Kim Campbell	M. Jodi Rell	Mary E Peters	Nadezhda Mihailova
Kim Guadagno	Mabel Vernon	Mary Elizabeth "Tipper" Gore	Nadine Gordimer
Kim Phuc	Madeleine Albright	Mary Fallin	Nadine Strossen
Kim Reynolds	Madeleine Bordallo	Mary Fisher	Nafis Sadik
Kim Weaver	Madeleine L'Engle	Mary Jane Coggeshall	Nan Hayworth
Kimberle Williams Crenshaw	Maggie Hassan	Mary Jo Kilroy	Nan Keohane
Kirsten E Gillibrand	Mairead Maguire	Mary L. Azcuenaga	Nancy A Dunkel
Kristi Noem	Malala Yousafzai	Mary Landrieu	Nancy Birdsall
Kyrsten Sinema	Marcia L Fudge	Mary O'Rourke	Nancy Gertner
Laura Bush	Marcia Pally	Mary P McAleese	Nancy Kassebaum Baker
Laura Chinchilla	Marcia S. Smith	Mary Pena	Nancy Lord
Laura D'Andrea Tyson	Marcy C Kaptur	Mary Reynolds	Nancy Pelosi
Laura Kelly	Margaret Atwood	Mary Robinson	Nancy Reagan
Laura Richardson	Margaret Chase Smith	Mary Taylor	Nancy Rubin
Laurie Marker	Margaret H Sanger	Mary-Claire King	Nancy W. Dickey
Lea Rabin	Margaret J. Geller	Maryam Elahi	Nancy Wyman
Lenora B Fulani	Margaret Spellings	Marye Anne Fox	Nanette Diaz Barragan
Lillian Vernon	Margaret Thatcher	Matilda Joslyn Gage	Nannie Helen Burroughs
Linda A. Suydam	Margaret Warner	Maxine Waters	Naomi Wolf
Linda Chavez-Thompson	Margaretha de Boer	Maya Angelou	Natalie Tennant
Linda K Kerber	Marguerite Rivera Houze	Mazie K Hirono	Nicole Fontaine
Linda Lingle	Mari Kiviniemi	Megan Barry	Niki Tsongas
Linda McMahon	Maria Cantwell	Meghan McCain	Nikki Haley
Linda S. Fink	Maria De Los Angeles Florez	Melania Trump	Nita M Lowey
Linda Sanchez	Maria Eva Peron	Melinda Brown	Nora Callahan
Linda Sarsour	Marian Wright Edelman	Melissa Bean	Nora Ephron
Linda Tarr-Whelan	Marianne Williamson	Meredith Monk	Norma Torres
Lisa Blunt Rochester	Mariannette Miller-Meeks	Mia Love	Nydia Velazquez
Lisa Murkowski	Marie Curie	Michaelle Jean	Olympia Jean Snowe
Lisa P Jackson	Marie-Andree Bertrand	Michele M Bachmann	Opal Tometi
Lois Capps	Marilyn Tucker Quayle	Michele Norris	Oprah Winfrey

Table B1 continued

Pam Iorio	S.E. Cupp	Susan C Schwab	Winona LaDuke
Pamela J. Parker	Sadako Ogata	Susan Collins	Wislawa Szymborska
Patricia A. McGuire	Sally Pederson	Susan Davis	Yulia Tymoshenko
Patricia Schroeder	Sally Quillian Yates	Susan E Rice	Yvette D Clarke
Patrisse Khan-Cullors	Sally Quinn	Susan Eckerly	Yvonne Prettner Solon
Patsy Mink	Sally Regenhard	Susan Esserman	Zoe Lofgren
Patty Judge	Samantha Power	Susan Faludi	Zuzana Caputova
Patty Murray	Sandy Adams	Susan Golding	
Paula Casey	Sandy Thurman	Susan Hammer	
Paula Treckel	Sarah Bain	Susan Molinari	
Paulina W. Davis	Sarah Brady	Susan Narvaiz	
Pauline Hanson	Sarah Palin	Susan Ness	
Pearl S. Buck	Sarah Parker Remond	Susana Martinez	
Phyllis Chesler	Shannon Lucid	Susanne Agnelli	
Phyllis E. Oakley	Shari Steele	Suzan DelBene	
Phyllis Greenberger	Sharon Sayles Belton	Suzanne Bonamici	
Phyllis Schlafly	Sheikh Hasina	Suzanne Crouch	
Polly Peachum	Sheila Jackson Lee	Suzanne M Kosmas	
Pratibha D Patil	Sheila Oliver	Takako Doi	
Queen Elizabeth I	Sheila Simon	Tamika Mallory	
Queen Elizabeth II	Sheila Widnall	Tammy Duckworth	
Queen Noor of Jordan	Shelley Berkley	Tammy S Baldwin	
Queen Raina of Jordan	Shelley L. Davis	Tanya Metaksa	
Queen Victoria	Shelley Moore Capito	Tarana Burke	
Rachel Carson	Shenna Bellows	Tarja K Halonen	
Rachelle Chong	Sherri W. Goodman	Tempe Herndon Durham	
Rebecca Kleefisch	Shirin Ebadi	Terri Lynn Land	
Rebecca L Felton	Shirley Chisholm	Terri Sewell	
Rebecca Michelle "Mikie" Sherrill	Shirley Franklin	Thelma Catherine "Pat" Nixon	
Regina C. Brown	Shirley M. Hufstedler	Theresa May	
Ren Volpe	Shirley M. Malcom	Tina Smith	
Renee Chelian	Sissela Bok	Toni Morrison	
Renee Ellmers	Sojourner Truth	Tsai Ing-wen	
Rigoberta Menchu Tum	Soledad O'Brien	Tulsi Gabbard	
Rita Levi-Montalcini	Somaia Barghouti	Urvashi Vaid	
Roberta L. Gross	Sonia Fuentes	Usha Narayanan	
Roberta R. Katz	Sonia Johnson	Vaira Vike-Freiberga	
Robin Abrams	Sonia Sotomayor	Valdez "Val" Demings	
Ronda Hauben	Stacey Abrams	Valentina Tereshkova	
Rosa L. DeLauro	Stacey Plaskett	Valerie Solanas	
Rosa Parks	Staci Appel	Vandana Shiva	
Rosalyn S. Yalow	Stephanie H Sandlin	Vesna Pesic	
Rosalynn Carter	Stephanie Murphy	Vicky Hartzler	
Rosario G. Manalo	Stephanie Tubbs Jones	Victoria C. Woodhull	
Roxanne Qualls	Sue Ellspermann	Virginia Foxx	
Roza Otunbayeva	Sue Minter	Wanda Wilk	
Ruth A Minner	Sue Suter	Wangari Maathai	
Ruth Bader Ginsburg	Susan Allen	Wendy Davis	
Ruth Mandel	Susan B. Anthony	Wendy Long	
Ruth Simmons	Susan Brooks	Wilma Mankiller	

Appendix C

LDA-Based Topic Modelling

In addition to the transformer model reported in the paper, we also calculated an LDA-based model for robustness. Specifically, we have calculated the Coherence score using our corpus for the LDA-based mCTM. Our coherence measure C_v uses a sliding window principle, considering a one-set segmentation of top verbal terms as well as a validation measure based on the normalized pointwise mutual information (NPMI) and the cosine similarity. Figure C1 below shows coherence scores for the number of topics 1 to 20. It demonstrates that the optimal number of topics is 17, yielding the highest coherence score of 0.421.

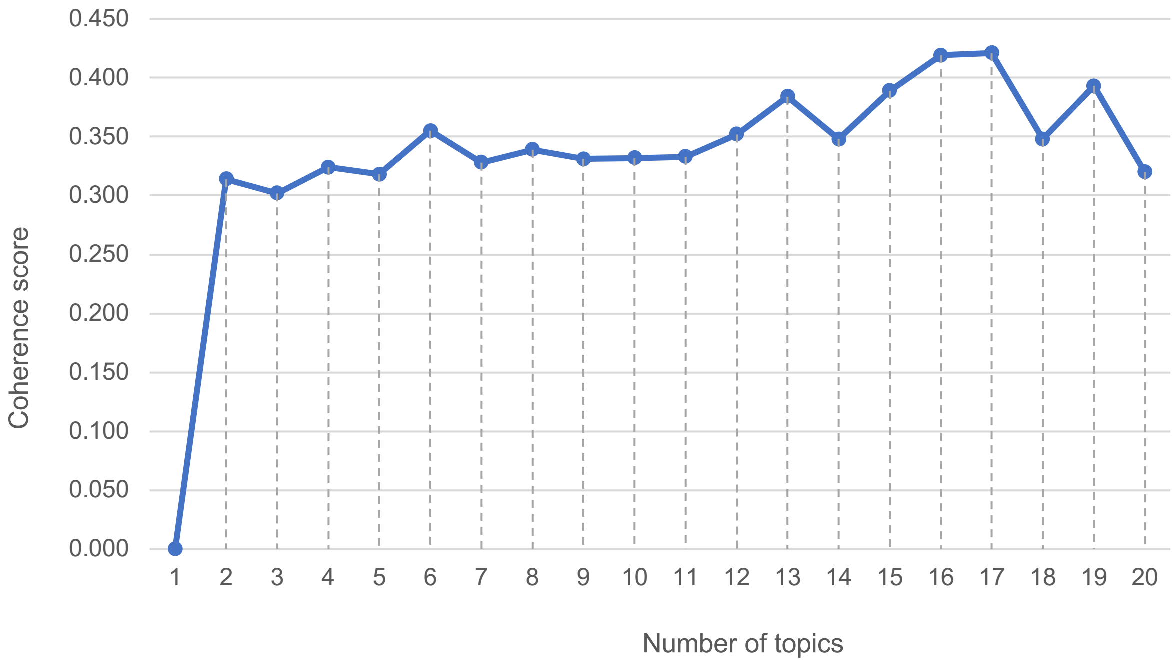

Figure C1 Coherence Score and Optimal Number of Topics

Our analysis also shows that all 17 topics are distinct with only minor overlaps present between them as demonstrated on Figure C2.

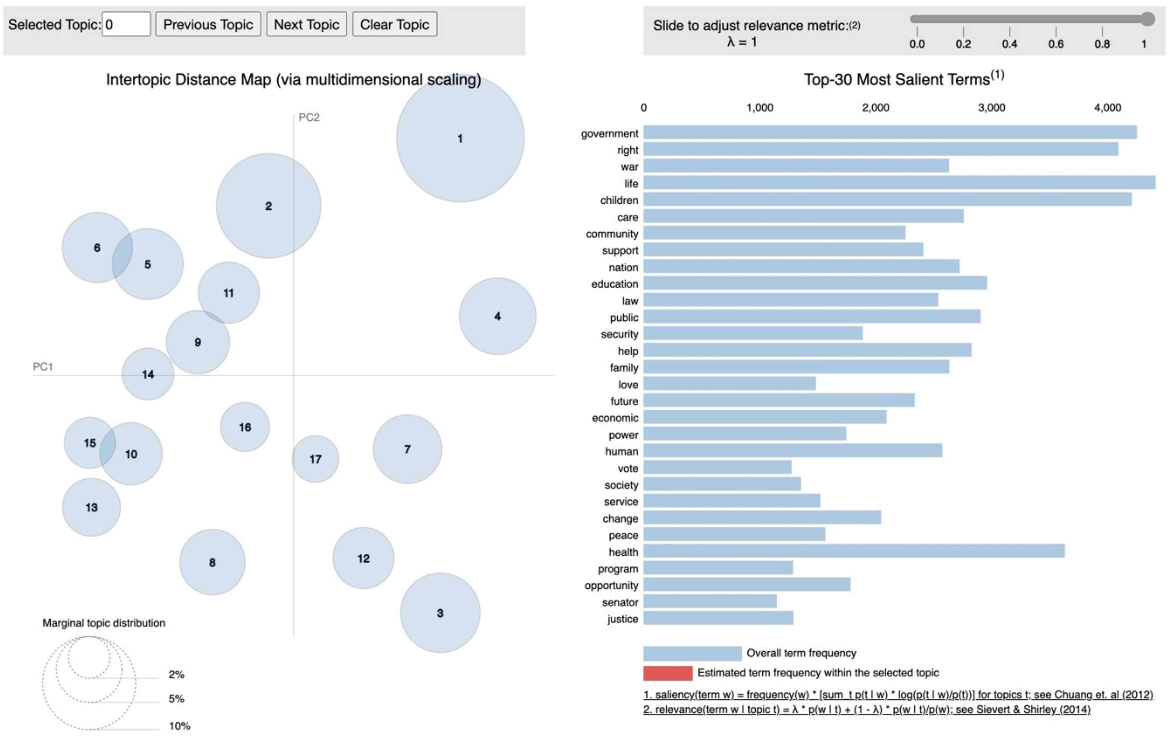

Figure C2 Inter-topic Distance Map

Appendix D

Table D1 Multilevel Regression Results

Topic 1:

	Coefficient	SE	t	P>t	[95% Conf. Interval]	
**A**	-0.0087	0.0185	-0.47	0.64	-0.0451	0.0276
**B**	0.0256	0.0151	1.7	0.09	-0.0040	0.0551
**H**	-0.0069	0.0215	-0.32	0.75	-0.0492	0.0353
**W**	0.0085	0.0132	0.64	0.52	-0.0174	0.0344
**Constant**	0.0344	0.0127	2.7	0.01	0.0094	0.0594
σ_u	0.0409					
σ_e	0.1141					
ρ	0.1136					
**p**	0.1129					

Topic 2:

	Coefficient	SE	t	P>t	[95% Conf. Interval]	
**A**	-0.0118176	0.0178437	-0.66	0.508	-0.0468198	0.0231847
**B**	-0.004678	0.014484	-0.32	0.747	-0.0330899	0.0237338
**H**	-0.0136192	0.0207159	-0.66	0.511	-0.0542556	0.0270171
**W**	0.0122197	0.0127169	0.96	0.337	-0.0127258	0.0371652
**Constant**	0.0194981	0.0122629	1.59	0.112	-0.0045568	0.043553
σ_u	0.0173485					
σ_e	0.10981818					
ρ	0.02434837					
**p**	0.0691					

Topic 3:

	Coefficient	SE	t	P>t	[95% Conf. Interval]	
**A**	-0.0174196	0.0153675	-1.13	0.257	-0.0475645	0.0127252
**B**	-0.0094516	0.012474	-0.76	0.449	-0.0339206	0.0150174
**H**	-0.0279376	0.0178411	-1.57	0.118	-0.0629347	0.0070594
**W**	-0.0022418	0.0109521	-0.2	0.838	-0.0237255	0.0192419
**Constant**	0.0320094	0.0105611	3.03	0.002	0.0112927	0.0527261
σ_u	0.02558392					
σ_e	0.09457814					
ρ	0.06818406					
**p**	0.2543					

Topic 4:

	Coefficient	SE	t	P>t	[95% Conf. Interval]	
**A**	0.0152418	0.015288	1	0.319	-0.0147471	0.0452307
**B**	-0.0002109	0.0124095	-0.02	0.986	-0.0245533	0.0241316
**H**	-0.0050962	0.0177488	-0.29	0.774	-0.0399122	0.0297198
**W**	0.017188	0.0108955	1.58	0.115	-0.0041846	0.0385605
**Constant**	0.0217688	0.0105065	2.07	0.038	0.0011593	0.0423784
σ_u	0.01844077					
σ_e	0.09408895					
ρ	0.03699225					
**p**	0.049					

Topic 5:

	Coefficient	SE	t	P>t	[95% Conf. Interval]	
**A**	0.0057467	0.0107466	0.53	0.593	-0.0153337	0.0268272
**B**	0.000486	0.0087231	0.06	0.956	-0.0166253	0.0175973
**H**	-0.0004875	0.0124764	-0.04	0.969	-0.0249611	0.0239862
**W**	0.0047462	0.0076589	0.62	0.536	-0.0102775	0.0197699
**Constant**	0.0290523	0.0073855	3.93	0	0.014565	0.0435396
σ_u	0.18905594					
σ_e	0.06613907					
ρ	0.89095826					
**p**	0.8658					

Topic 6:

	Coefficient	SE	t	P>t	[95% Conf. Interval]	
**A**	-0.0209618	0.0127969	-1.64	0.102	-0.0460641	0.0041404
**B**	-0.0221644	0.0103874	-2.13	0.033	-0.0425402	-0.0017885
**H**	-0.0332112	0.0148567	-2.24	0.026	-0.062354	-0.0040684
**W**	-0.0119146	0.0091201	-1.31	0.192	-0.0298045	0.0059754
**Constant**	0.038803	0.0087945	4.41	0	0.0215517	0.0560543
σ_u	0.01155267					
σ_e	0.07875739					
ρ	0.0210638					
**p**	0.0721					

Topic 7:

	Coefficient	SE	t	P>t	[95% Conf. Interval]	
**A**	0.0041188	0.0129274	0.32	0.75	-0.0212396	0.0294772
**B**	0.0152631	0.0104934	1.45	0.146	-0.0053207	0.0358469
**H**	0.0020082	0.0150083	0.13	0.894	-0.0274319	0.0314484
**W**	0.0097326	0.0092132	1.06	0.291	-0.00834	0.0278051
**Constant**	0.0222846	0.0088842	2.51	0.012	0.0048574	0.0397119
σ_u	0.15155607					
σ_e	0.07956099					
ρ	0.78395477					
**p**	0.5678					

Topic 8:

	Coefficient	SE	t	P>t	[95% Conf. Interval]	
**A**	-0.0108702	0.0089213	-1.22	0.223	-0.0283703	0.0066299
**B**	-0.0033905	0.0072416	-0.47	0.64	-0.0175956	0.0108146
**H**	-0.0059708	0.0103573	-0.58	0.564	-0.0262878	0.0143461
**W**	-0.0075488	0.0063581	-1.19	0.235	-0.0200208	0.0049232
**Constant**	0.02368	0.0061311	3.86	0	0.0116532	0.0357067
σ_u	0.00741713					
σ_e	0.05490579					
ρ	0.01792177					
**p**	0.6132					

Topic 9:

	Coefficient	SE	t	P>t	[95% Conf. Interval]	
**A**	0.0000031	0.0108916	0	1	-0.0213618	0.021368
**B**	-0.0007285	0.0088408	-0.08	0.934	-0.0180707	0.0166137
**H**	0.0150483	0.0126447	1.19	0.234	-0.0097556	0.0398521
**W**	0.0025286	0.0077622	0.33	0.745	-0.0126978	0.017755
**Constant**	0.0126029	0.0074851	1.68	0.092	-0.0020799	0.0272857
σ_u	0.02585957					
σ_e	0.06703146					
ρ	0.1295478					
**p**	0.6981					

Topic 10:

	Coefficient	SE	t	P>t	[95% Conf. Interval]	
**A**	-0.0021607	0.0094925	-0.23	0.82	-0.0207812	0.0164598
**B**	0.0074235	0.0077052	0.96	0.335	-0.0076911	0.022538
**H**	0.0005841	0.0110205	0.05	0.958	-0.0210336	0.0222018
**W**	0.0075998	0.0067652	1.12	0.261	-0.0056708	0.0208703
**Constant**	0.0138962	0.0065236	2.13	0.033	0.0010995	0.026693
σ_u	0.00900854					
σ_e	0.05842109					
ρ	0.02322542					
**p**	0.4977					

Topic 11:

	Coefficient	SE	t	P>t	[95% Conf. Interval]	
**A**	0.0021359	0.0117896	0.18	0.856	-0.0209906	0.0252624
**B**	0.0069491	0.0095698	0.73	0.468	-0.011823	0.0257212
**H**	0.007286	0.0136873	0.53	0.595	-0.019563	0.034135
**W**	0.0120188	0.0084023	1.43	0.153	-0.0044631	0.0285006
**Constant**	0.006579	0.0081023	0.81	0.417	-0.0093144	0.0224724
σ_u	0.00996877					
σ_e	0.07255846					
ρ	0.01852619					
**p**	0.4638					

Topic 12:

	Coefficient	SE	t	P>t	[95% Conf. Interval]	
**A**	0.0452459	0.0118724	3.81	0	0.0219571	0.0685348
**B**	0.0029508	0.009637	0.31	0.76	-0.0159531	0.0218546
**H**	0.0003623	0.0137834	0.03	0.979	-0.0266751	0.0273998
**W**	-0.0035233	0.0084612	-0.42	0.677	-0.0201209	0.0130742
**Constant**	0.0199445	0.0081592	2.44	0.015	0.0039395	0.0359495
σ_u	0.03001355					
σ_e	0.07306773					
ρ	0.14436787					
**p**	0.0000					

Topic 13:

	Coefficient	SE	t	P>t	[95% Conf. Interval]	
**A**	0.0004617	0.0089025	0.05	0.959	-0.0170015	0.0179248
**B**	0.0061515	0.0072263	0.85	0.395	-0.0080236	0.0203266
**H**	-0.0009924	0.0103355	-0.1	0.924	-0.0212665	0.0192817
**W**	0.0084757	0.0063447	1.34	0.182	-0.00397	0.0209214
**Constant**	0.0121652	0.0061182	1.99	0.047	0.0001638	0.0241665
σ_u	0.00792814					
σ_e	0.05478995					
ρ	0.02050885					
**p**	0.4003					

Topic 14:

	Coefficient	SE	t	P>t	[95% Conf. Interval]	
**A**	0.0061126	0.0061481	0.99	0.32	-0.0059474	0.0181727
**B**	-0.0001556	0.0049905	-0.03	0.975	-0.0099449	0.0096338
**H**	-0.0033688	0.0071377	-0.47	0.637	-0.0173701	0.0106326
**W**	0.0010863	0.0043816	0.25	0.804	-0.0075087	0.0096813
**Constant**	0.017968	0.0042252	4.25	0	0.0096798	0.0262561
σ_u	0.01123702					
σ_e	0.03783802					
ρ	0.08104732					
**p**	0.7141					

Topic 15:

	Coefficient	SE	t	P>t	[95% Conf. Interval]	
**A**	0.0190764	0.0133425	1.43	0.153	-0.0070963	0.0452491
**B**	0.0029007	0.0108303	0.27	0.789	-0.018344	0.0241455
**H**	0.0038842	0.0154902	0.25	0.802	-0.0265014	0.0342697
**W**	0.0125653	0.009509	1.32	0.187	-0.0060875	0.0312182
**Constant**	0.0122206	0.0091695	1.33	0.183	-0.0057663	0.0302075
σ_u	0.03037847					
σ_e	0.08211576					
ρ	0.12038473					
**p**	0.2959					

Topic 16:

	Coefficient	SE	t	P>t	[95% Conf. Interval]	
**A**	-0.0016172	0.0109108	-0.15	0.882	-0.0230198	0.0197854
**B**	0.0020345	0.0088565	0.23	0.818	-0.0153384	0.0194073
**H**	0.0646801	0.0126671	5.11	0	0.0398324	0.0895278
**W**	0.0055443	0.007776	0.71	0.476	-0.009709	0.0207976
**Constant**	0.0137628	0.0074983	1.84	0.067	-0.000946	0.0284715
σ_u	0.01374357					
σ_e	0.06714993					
ρ	0.04020556					
**p**	0.0000					

Topic 17:

	Coefficient	SE	t	P>t	[95% Conf. Interval]	
A	0.0094048	0.0095172	0.99	0.323	-0.0092641	0.0280737
B	-0.0071462	0.0077252	-0.93	0.355	-0.0223	0.0080076
H	-0.0114792	0.0110491	-1.04	0.299	-0.0331531	0.0101946
W	-0.0097353	0.0067827	-1.44	0.151	-0.0230403	0.0035697
Constant	0.0268582	0.0065406	4.11	0	0.0140283	0.0396882
σ_u	0.00800149					
σ_e	0.05857285					
ρ	0.01831971					
p	0.0699					

Topic 18:

	Coefficient	SE	t	P>t	[95% Conf. Interval]	
A	0.0032789	0.0097464	0.34	0.737	-0.0158397	0.0223976
B	-0.0091749	0.0079113	-1.16	0.246	-0.0246938	0.0063439
H	-0.0109939	0.0113153	-0.97	0.331	-0.03319	0.0112021
W	-0.0083523	0.0069461	-1.2	0.229	-0.0219778	0.0052732
Constant	0.0264038	0.0066981	3.94	0	0.0132647	0.0395428
σ_u	0.03420509					
σ_e	0.05998386					
ρ	0.24538078					
p	0.4124					

Topic 19:

	Coefficient	SE	t	P>t	[95% Conf. Interval]	
A	0.0309452	0.0067927	4.56	0	0.0176206	0.0442698
B	0.010586	0.0055138	1.92	0.055	-0.0002298	0.0214017
H	0.0202071	0.0078861	2.56	0.01	0.0047377	0.0356764
W	0.003718	0.0048411	0.77	0.443	-0.0057782	0.0132143
Constant	0.014119	0.0046682	3.02	0.003	0.0049618	0.0232762
σ_u	0.0137481					
σ_e	0.04180539					
ρ	0.09759401					
p	0.0000					

Topic 20:

	Coefficient	SE	t	P>t	[95% Conf. Interval]	
A	0.0013649	0.0109235	0.12	0.901	-0.0200626	0.0227925
B	0.0047017	0.0088668	0.53	0.596	-0.0126913	0.0220947
H	0.0154938	0.0126818	1.22	0.222	-0.0093828	0.0403703
W	0.0115359	0.007785	1.48	0.139	-0.0037352	0.026807
Constant	0.010944	0.0075071	1.46	0.145	-0.0037818	0.0256698
σ_u	0.01091031					
σ_e	0.06722803					
ρ	0.02566157					
p	0.2854					

Topic 21:

	Coefficient	SE	t	P>t	[95% Conf. Interval]	
A	0.0012727	0.0093474	0.14	0.892	-0.0170631	0.0196085
B	-0.0004741	0.0075874	-0.06	0.95	-0.0153576	0.0144093
H	-0.0044569	0.010852	-0.41	0.681	-0.0257441	0.0168303
W	0.006162	0.0066617	0.92	0.355	-0.0069056	0.0192296
Constant	0.0126828	0.0064239	1.97	0.049	0.0000818	0.0252839
σ_u	0.00968183					
σ_e	0.05752783					
ρ	0.0275441					
p	0.3741					

Topic 22:

	Coefficient	SE	t	P>t	[95% Conf. Interval]	
A	0.0010987	0.0104856	0.1	0.917	-0.0194699	0.0216672
B	0.0175852	0.0085113	2.07	0.039	0.0008893	0.034281
H	0.0054026	0.0121734	0.44	0.657	-0.0184767	0.029282
W	0.0124156	0.0074729	1.66	0.097	-0.0022433	0.0270744
Constant	0.0075454	0.0072061	1.05	0.295	-0.0065901	0.0216809
σ_u	0.02279568					
σ_e	0.06453309					
ρ	0.11093618					
p	0.1517					

Topic 23:

	Coefficient	SE	t	P>t	[95% Conf. Interval]	
A	-0.0060235	0.0100895	-0.6	0.551	-0.025815	0.013768
B	-0.0005327	0.0081898	-0.07	0.948	-0.0165977	0.0155323
H	-0.0139343	0.0117135	-1.19	0.234	-0.0369114	0.0090429
W	-0.0070267	0.0071906	-0.98	0.329	-0.0211317	0.0070784
Constant	0.021967	0.0069339	3.17	0.002	0.0083655	0.0355685
σ_u	0.08403821					
σ_e	0.06209493					
ρ	0.64684839					
p	0.489					

Topic 24:

	Coefficient	SE	t	P>t	[95% Conf. Interval]	
A	0.0063183	0.0081099	0.78	0.436	-0.00959	0.0222267
B	-0.0028008	0.0065829	-0.43	0.671	-0.0157139	0.0101123
H	-0.0030539	0.0094153	-0.32	0.746	-0.0215229	0.0154152
W	-0.0012486	0.0057798	-0.22	0.829	-0.0125862	0.0100891
Constant	0.0220874	0.0055734	3.96	0	0.0111545	0.0330203
σ_u	0.13174798					
σ_e	0.04991194					
ρ	0.87449064					
p	0.7566					

Topic 25:

	Coefficient	SE	t	P>t	[95% Conf. Interval]	
**A**	0.0060186	0.0080432	0.75	0.454	-0.0097589	0.0217961
**B**	0.0067111	0.0065288	1.03	0.304	-0.0060957	0.0195179
**H**	0.000454	0.0093378	0.05	0.961	-0.017863	0.0187711
**W**	0.0060902	0.0057322	1.06	0.288	-0.0051541	0.0173345
**Constant**	0.0144475	0.0055276	2.61	0.009	0.0036046	0.0252904
σ_u	0.0889037					
σ_e	0.04950116					
ρ	0.76334654					
**p**	0.7874					

Topic 26:

	Coefficient	SE	t	P>t	[95% Conf. Interval]	
**A**	-0.0349834	0.009693	-3.61	0	-0.0539971	-0.0159697
**B**	-0.0267237	0.0078679	-3.4	0.001	-0.0421575	-0.01129
**H**	-0.0342112	0.0112532	-3.04	0.002	-0.0562854	-0.012137
**W**	-0.0262489	0.006908	-3.8	0	-0.0397997	-0.0126982
**Constant**	0.047118	0.0066614	7.07	0	0.0340511	0.060185
σ_u	0.01330718					
σ_e	0.05965473					
ρ	0.04740149					
**p**	0.0015					

Topic 27:

	Coefficient	SE	t	P>t	[95% Conf. Interval]	
**A**	-0.0120717	0.0094421	-1.28	0.201	-0.0305932	0.0064499
**B**	-0.0182831	0.0076643	-2.39	0.017	-0.0333173	-0.003249
**H**	-0.0255746	0.0109619	-2.33	0.02	-0.0470774	-0.0040718
**W**	-0.0201688	0.0067292	-3	0.003	-0.0333688	-0.0069688
**Constant**	0.0384037	0.0064889	5.92	0	0.0256749	0.0511324
σ_u	0.01343142					
σ_e	0.0581105					
ρ	0.05071445					
**p**	0.0347					

Topic 28:

	Coefficient	SE	t	P>t	[95% Conf. Interval]	
**A**	0.0395336	0.0113048	3.5	0	0.0173581	0.0617091
**B**	0.0137237	0.0091763	1.5	0.135	-0.0042765	0.0317239
**H**	0.0338795	0.0131245	2.58	0.01	0.0081345	0.0596244
**W**	0.0102678	0.0080568	1.27	0.203	-0.0055363	0.026072
**Constant**	0.0078167	0.0077691	1.01	0.315	-0.0074232	0.0230565
σ_u	0.0121416					
σ_e	0.06957476					
ρ	0.02955423					
**p**	0.001					

Topic 29:

	Coefficient	SE	t	P>t	[95% Conf. Interval]	
A	-0.0079871	0.0119039	-0.67	0.502	-0.0313379	0.0153636
B	0.0045065	0.0096626	0.47	0.641	-0.0144477	0.0234606
H	-0.014082	0.01382	-1.02	0.308	-0.0411913	0.0130273
W	-0.0019397	0.0084837	-0.23	0.819	-0.0185813	0.014702
Constant	0.0223062	0.0081808	2.73	0.006	0.0062587	0.0383537
σ_u	0.00911434					
σ_e	0.07326193					
ρ	0.01524135					
p	0.4993					

Topic 30:

	Coefficient	SE	t	P>t	[95% Conf. Interval]	
A	-0.0177227	0.010524	-1.68	0.092	-0.0383666	0.0029212
B	-0.0176349	0.0085425	-2.06	0.039	-0.0343919	-0.000878
H	-0.0194844	0.012218	-1.59	0.111	-0.0434512	0.0044824
W	-0.0153784	0.0075003	-2.05	0.041	-0.0300909	-0.0006658
Constant	0.0307093	0.0072325	4.25	0	0.016522	0.0448966
σ_u	0.00879758					
σ_e	0.06476945					
ρ	0.01811533					
p	0.2885					

Topic 31:

	Coefficient	SE	t	P>t	[95% Conf. Interval]	
A	0.000427	0.0069309	0.06	0.951	-0.0131686	0.0140226
B	0.0022947	0.0056259	0.41	0.683	-0.008741	0.0133305
H	0.0006867	0.0080465	0.09	0.932	-0.0150973	0.0164707
W	0.0062226	0.0049395	1.26	0.208	-0.0034667	0.015912
Constant	0.0111115	0.0047632	2.33	0.02	0.0017681	0.0204549
σ_u	0.01312592					
σ_e	0.04265561					
ρ	0.08649997					
p	0.3966					

Topic 32:

	Coefficient	SE	t	P>t	[95% Conf. Interval]	
A	-0.0228052	0.0089911	-2.54	0.011	-0.0404422	-0.0051682
B	-0.0237924	0.0072982	-3.26	0.001	-0.0381086	-0.0094762
H	-0.0279599	0.0104384	-2.68	0.007	-0.0484358	-0.007484
W	-0.0226082	0.0064078	-3.53	0	-0.0351778	-0.0100386
Constant	0.0406079	0.0061791	6.57	0	0.0284871	0.0527287
σ_u	0.00881952					
σ_e	0.05533535					
ρ	0.02477364					
p	0.0095					

Topic 33:

	Coefficient	SE	t	P>t	[95% Conf. Interval]	
A	-0.0012388	0.0116715	-0.11	0.915	-0.0241336	0.021656
B	0.0347934	0.0094739	3.67	0	0.0162093	0.0533774
H	0.0016757	0.0135502	0.12	0.902	-0.0249043	0.0282558
W	0.0061841	0.0083181	0.74	0.457	-0.0101327	0.0225008
Constant	0.015009	0.0080211	1.87	0.062	-0.0007253	0.0307432
σ_u	0.02609828					
σ_e	0.07183162					
ρ	0.11661213					
p	0.0000					

Topic 34:

	Coefficient	SE	t	P>t	[95% Conf. Interval]	
A	-0.0010396	0.0055647	-0.19	0.852	-0.0119553	0.0098762
B	0.0036688	0.004517	0.81	0.417	-0.0051916	0.0125293
H	-0.0053745	0.0064604	-0.83	0.406	-0.0180473	0.0072983
W	0.0014463	0.0039659	0.36	0.715	-0.0063332	0.0092257
Constant	0.0141323	0.0038243	3.7	0	0.0066306	0.021634
σ_u	0.0061823					
σ_e	0.03424771					
ρ	0.0315581					
p	0.5348					

Topic 35:

	Coefficient	SE	t	P>t	[95% Conf. Interval]	
A	0.000515	0.0075756	0.07	0.946	-0.0143452	0.0153752
B	0.0024042	0.0061492	0.39	0.696	-0.009658	0.0144665
H	0.0474069	0.0087949	5.39	0	0.0301548	0.0646591
W	0.0025999	0.005399	0.48	0.63	-0.0079907	0.0131905
Constant	0.0154413	0.0052062	2.97	0.003	0.0052288	0.0256538
σ_u	0.08403941					
σ_e	0.04662324					
ρ	0.76465521					
p	0.0000					

Notes: In all regression, the number of observations was 1,599. Panel effects were measured at the level of each year and each individual within a year.

Appendix E

Table E1 Standard Deviations in Behavioral Schemas by Group

Behavioral Schema=Topic	SD (B)	SD(W)	SD(A)	SD(H)
Future and nation	0.13215539	0.07096267	0.02641084	0.10643747
Environment, sustainability, energy, and water	0.03104003	0.10848524	0.0166035	0.00397762
Healthcare	0.06102255	0.08928849	0.00798762	0.02452031
Jobs, economy, and employment	0.0401674	0.08092493	0.06967997	0.02518888
Women’s rights	0.05374145	0.07853714	0.01425212	0.0066645
Budget, public policy, government, and taxes	0.04163614	0.04071339	0.01080844	0.01203942
Diversity, race, gender, and childhood opportunities	0.11984762	0.08930144	0.0172355	0.00590514
Domestic violence	0.03039594	0.05044836	0.00410313	0.05376326
Pregnancy, abortion, and women's right to choose	0.05174054	0.0728277	0.0370223	0.09550122
Life and education	0.01369916	0.02515177	0.00457503	0.02007338
Cyber security, internet technology	0.00563853	0.10507573	0.00501471	0.00523821
Refugees and humanitarian action	0.04014463	0.06421017	0.07956091	0.01631757
Higher education	0.01237452	0.05346389	0.00408097	0.02382805
War and nuclear weapons	0.0144685	0.02491957	0.01318286	0.01081873
Justice	0.01234232	0.10074098	0.02277286	0.01760659
Defense and military	0.0084749	0.03657214	0.00633999	0.09941534
Europe, integration, European Union	0.02362982	0.04941613	0.07482441	0.02352022
Peace and regional conflict	0.01755909	0.03283828	0.01987115	0.01082456
Diplomacy	0.01774451	0.0300059	0.04285244	0.04930223
Public education and children	0.00932149	0.08372629	0.0055778	0.05471441
Art and music	0.0064378	0.05171165	0.02080816	0.00742223
Cities and neighborhoods	0.10096303	0.06958476	0.00447554	0.00695796
HIV, AIDS	0.1015179	0.07790498	0.00941975	0.01122559
United Nations and human rights	0.01080196	0.07621577	0.01383377	0.00737286
Love and family	0.01335262	0.06824225	0.03076132	0.01139371
Disability	0.01363561	0.03638535	0.005183	0.00824454
Science and technology	0.03554556	0.05296937	0.07072794	0.00753775
Development and poverty	0.10189973	0.0515706	0.06597524	0.04517742
Law	0.05264908	0.06512539	0.00887556	0.00727487
Space	0.00666454	0.08078904	0.00641349	0.00854833
Mental health and self-help	0.00649349	0.05448193	0.00470758	0.00527616
Army and military service	0.00891627	0.01614034	0.01172354	0.00861587
Rights of black community	0.11102	0.06182198	0.00847849	0.01087088
Chronic debt	0.03393534	0.01716561	0.0054642	0.00707651
Labor union and workers	0.01290924	0.02480253	0.00655474	0.19945119

Notes: B- female leaders with any Black background; W – female leaders with any White background; A- female leaders with any Asian background; H – female leaders with any Hispanic/Latino background. In the paper, we concentrate on black female leaders. Results for Asian and Hispanic/Latino groups are included to demonstrate that the results are similar to those obtained for the Black background group (though sample sizes in those groups are significantly smaller as reported in the paper).
